# Heavy-tailed update distributions arise from information-driven self-organization in nonequilibrium learning

**DOI:** 10.1073/pnas.2523012122

**Published:** 2025-12-18

**Authors:** Xin-Ya Zhang, Chao Tang

**Affiliations:** ^a^Center for Interdisciplinary Studies and Department of Physics, School of Science, Westlake University, Hangzhou 310030, People’s Republic of China; ^b^Institute of Natural Sciences, Westlake Institute for Advanced Study, Hangzhou 310024, People’s Republic of China

**Keywords:** statistical physics, deep learning, self-organization criticality

## Abstract

Like human decision-making under real-world constraints, artificial neural networks may balance free exploration in parameter space with task-relevant adaptation. In this study, we identify consistent signatures of criticality during neural network training and provide theoretical evidence that such scaling behavior arises naturally from information-driven self-organization: a dynamic balance between the maximum entropy principle that promotes unbiased exploration and mutual information constraint that relates updates with task objective. We numerically demonstrate that the power-law exponent of updates remains stable throughout training, supporting the presence of self-organized criticality. Furthermore, we show that the loss landscape exhibits exponential ruggedness under small perturbations, transitioning to power-law ruggedness at larger scales, in the absence of mini-batch noise, indicating an intrinsic geometric landscape. We also observe a power-law distribution in the intervals between large updates, indicating an intermittent learning process. Together, these findings suggest that neural network learning reflects a nonequilibrium process governed by the fundamental trade-off between randomness and relevance, highlighting its dynamic nature and offering insights into the interpretability of AI systems.

## Significance

Artificial neural networks can adapt to tasks while freely exploring possible solutions, similar to how humans balance curiosity with goal-driven behavior. We show that during training, such networks naturally operate near a critical state. This state emerges from a balance between randomness and task relevance and leaves measurable signatures, including stable power-law statistics in parameter updates and multiscale patterns in the geometry of the loss landscape. Our findings reveal that neural network learning is a nonequilibrium process shaped by fundamental statistical principles, offering a general explanation for scaling laws in parameter updates and guiding the design of more interpretable and efficient intelligent systems.

Artificial neural networks (ANNs) have revolutionized the field of AI, driving remarkable advances across a wide range of domains, including computer vision, natural language processing, and scientific discovery (1–4). In these neural networks, trained weights serve as key repositories of learned knowledge, extracting complex yet classifiable patterns from training data (5–10). Extensive research has focused on the initialization and final configurations of parameters in neural network models (11–14). However, a comparatively underexplored aspect is the dynamics of parameter updates, the iterative adjustments that drive the transition from a randomly initialized model into one capable of performing a given task. These updates encapsulate the evolving interaction between model representation and external data during training (15–17). Exploring the statistical properties of updates across scales may reveal fundamental principles underlying how neural networks acquire and compress knowledge and provide a foundation for understanding and improving the transparency of learning processes in neural networks.

While recent work has reported signs of criticality in ANNs, analyses primarily rely on indirect or low-dimensional metrics. For example, prior studies have examined Hessian spectrum decompositions (18) and principal component analysis (PCA) of training trajectories (19), implicitly assuming a quasi-equilibrium condition (20, 21). However, neural network training proceeds via gradient descent-based optimization, which is inherently far from equilibrium (22, 23). The continuous injection of information and dynamic reorganization of network parameters suggest that learning is more faithfully described as a nonequilibrium process.

Consider a human decision-making process such as job searching. In an unconstrained scenario, individuals could explore all available job opportunities freely. Due to practical constraints such as resources and geographic restrictions, individuals are required to refine their decision by prioritizing some possibilities over others. Behavioral studies have shown that humans tend to combine directed and random exploration strategies depending on task horizon and perceived opportunity (24, 25). More generally, such a balance between randomness and constraint may give rise to an underlying critical regime (26–31), characterized by scaling laws. For instance, power-law-like cascading failures in earthquakes indicate the interaction between driving force and dissipation in self-organized criticality (32), while heavy-tailed neuronal connectivity arises from a trade-off between preferential and random growth under Hebbian self-organization (33).

Motivated by the parallels between physical and biological systems, we turn to the training of ANNs under nonequilibrium learning conditions. In this study, we investigate the emergence of criticality and the trade-offs that underlie learning in ANNs. Our analysis reveals that the magnitudes of the full parameter updates, measured without any dimensionality reduction, consistently exhibit heavy-tailed behavior across training stages, architectures, and hyperparameter configurations. These consistent patterns raise a central question: Do they reflect a general principle that governs the learning dynamics?

We propose that neural network learning operates as a nonequilibrium process shaped by information-driven self-organization. Specifically, we introduce a dual-factor framework based on first principles, where the observed scaling behavior arises from the combined effects of the maximum entropy principle and mutual information constraint. The maximum entropy principle ensures that parameter updates remain maximally unbiased under the current state of knowledge (i.e., training data), promoting random exploration. In contrast, the mutual information constraint introduces task relevance by favoring updates that maintain or enhance the dependency between inputs and task-relevant activations. Together, these two factors naturally give rise to a scale-free update distribution in far-from-equilibrium systems.

We further provide empirical support for this information-driven self-organization by demonstrating that the estimated power-law exponent remains stable throughout training. In addition, we observe a competing relationship between mutual information and entropy across training steps, consistent with the trade-off assumption in our theoretical formulation. To isolate the effect of mini-batch noise, we perform perturbation-based analyses probing the inherent geometric landscape through full-batch evaluations. Our result reveals multiscale ruggedness in the loss landscape: exponential decay under small perturbations that transitions to power-law scaling for large perturbations. Additionally, we observe power-law scaling in the temporal intervals between large parameter updates, indicating nonrandom timing patterns characteristic of critical dynamics in self-organizing systems.

## Results

### Heavy-Tailed Updates Across Training Stages.

Neural networks are typically initialized randomly and optimized using algorithms such as vanilla stochastic gradient descent (SGD), which guide them toward local minima. To investigate the underlying learning dynamics, we analyzed the full parameter updates across diverse neural network architectures and training stages. Our findings suggest that heavy-tailed updates consistently emerge throughout training, across different architectures and datasets, indicating a general principle underlying neural learning dynamics that transcends specific implementation details.

**Fig. 1.**
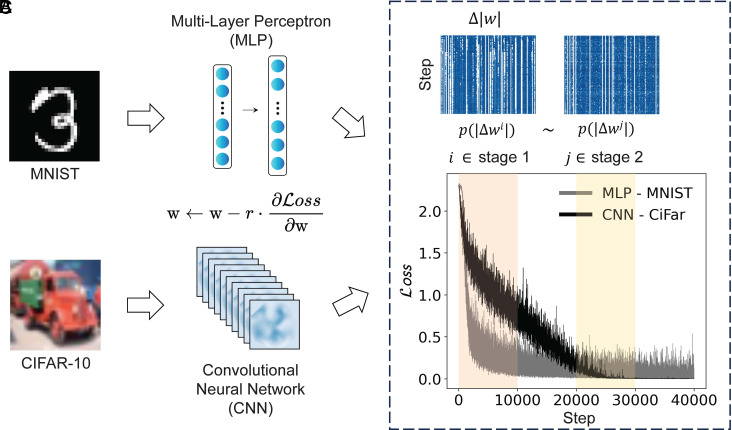
Overview of training tasks, model architectures, and loss dynamics. (*A*) Classification tasks are performed on the MNIST and CIFAR-10 datasets. The MNIST dataset consists of grayscale images of handwritten digits from 0 to 9, while CIFAR-10 contains color images across ten object categories. (*B*) Multilayer perceptron (MLP) and convolutional neural network (CNN) architectures are used for MNIST and CIFAR-10, respectively. The MLP consists of multiple fully connected layers with varying numbers of neurons (or units). The CNN is designed to extract hierarchical features from multichannel color images. Both models are trained using stochastic gradient descent with learning rate r. (*C*) Illustration of training loss dynamics over mini-batch steps. Despite different phases of training, e.g., stage 1 representing rapid loss decrease (steps 0 to 10,000), and stage 2 corresponding to near convergence (steps 20,000 to 30,000), the magnitude of updates |Δw| exhibits qualitatively similar patterns. Stages 1 and 2 correspond to fixed 10,000-iteration intervals selected to represent the early improvement regime and the later near-converged regime of training. During the near-convergence stage, mini-batch sampling can cause loss fluctuations, which was less pronounced in CNN due to the batch normalization employed. Despite differences in absolute loss values across architectures and tasks, both intervals illustrate the similar qualitative transition from rapid loss reduction to near-converged behavior. The *Lower* panel shows the evolution of cross-entropy loss for both MLP and CNN models, with shaded regions indicating the time windows selected for further analysis.

Specifically, we trained neural networks on two widely used image classification benchmarks: MNIST and CIFAR-10 (Fig. 1*A*; dataset information is provided in *Materials and Methods*). The multilayer perceptrons (MLPs) were trained on the grayscale MNIST dataset (digits 0 to 9, 10 classes), while the convolutional neural networks (CNNs) were trained on the RGB CIFAR-10 dataset (including 10 classes such as cars and airplanes). All neural network models were trained using SGD with a cross-entropy loss function and a mini-batch size of 64 and employed the Rectified Linear Unit (ReLU) activation function by default (Fig. 1*B*; see *Materials and Methods* for neural network training).

**Fig. 2.**
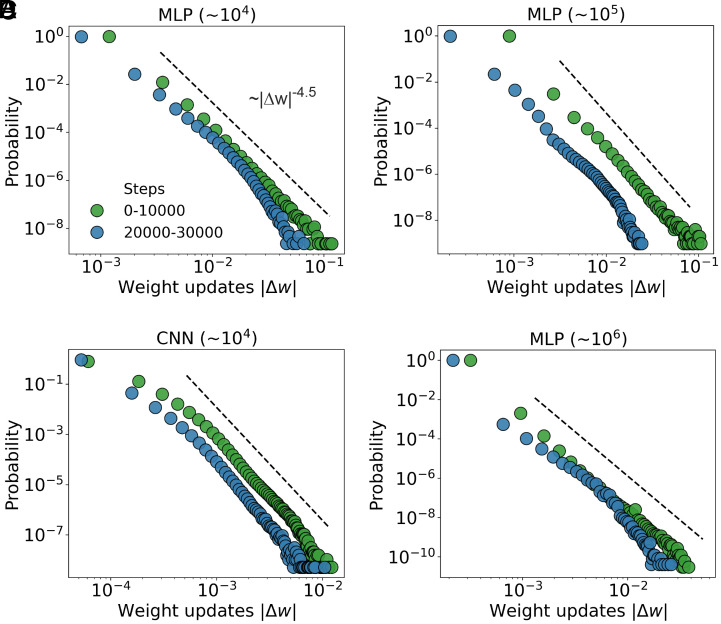
Heavy-tailed distributions of update magnitudes across models, scales, and training stages. Empirical probability distributions of absolute updates |Δw| are shown on a log–log scale for different model architectures and parameter scales (up to one million parameters). Green and blue dots correspond to different training stages as selected in Fig. 1: steps 0 to 10,000 (rapid loss decrease) and steps 20,000 to 30,000 (near convergence), respectively. (*A*) MLPs with approximately $10^{4}$ parameters (learning rate r=0.1) exhibits a heavy-tailed distribution with an indicative power-law slope of ${\left|\Delta w\right|}^{-4.5}$. (*B*) CNNs with approximately $10^{4}$ parameters (r=0.01) trained on CIFAR-10 shows similar heavy-tailed behavior. (*C* and *D*) MLPs with $10^{5}$ and $10^{6}$ parameters (see *Materials and Methods* for architecture details, r=0.1) further confirm the persistence of heavy-tailed update distributions at larger model scales, consistent with the indicative slopes observed in (*A* and *B*). Across all configurations, the distributions exhibit heavy-tailed behavior in both early and late training stages, suggesting a scale-invariant statistical property of the update dynamics throughout training. The flattening of the far tail results from a statistical sampling effect due to the rarity of large updates, as illustrated in the complementary cumulative distribution functions (*SI Appendix*, Fig. S1).

To examine whether the distribution of updates exhibits consistent behavior throughout training, we tracked the magnitude of parameter updates at each mini-batch step (Fig. 1*C*). We evaluated networks of varying sizes, spanning from $10^{4}$ to $10^{6}$ trainable parameters. Across different architectures (MLP and CNN), datasets, and training stages including both the rapid loss reduction phase (steps 0 to 10,000) and the later near convergence phase (steps 20,000 to 30,000), we consistently observed that the distribution of updates exhibits a heavy-tailed form:

[1] $$\begin{array}{c} p\left(\Delta w\right)\propto {\left|\Delta w\right|}^{-\alpha }, \end{array}$$

where α characterizes the heavy-tailedness of the power-law-like distribution. As illustrated in Fig. 2, a reference line with exponent α=4.5 is included as a visual guide to the observed heavy-tailed scaling. While the precise best-fit value of α may vary across conditions (e.g., ranging from 3 to 5, as shown in Fig. 2 and *SI Appendix*, Figs. S2–S5), the consistent emergence of heavy-tailed characteristics remains.

To assess the generality of the observed heavy-tailed behavior, we systematically varied several training configurations, including mini-batch size (*SI Appendix*, Fig. S2 *A* and *B*), learning rate (*SI Appendix*, Fig. S2*D*), later and more stable training phases (e.g., steps 80,000 to 90,000; *SI Appendix*, Fig. S2*C*), loss function (e.g., Mean Squared Error in *SI Appendix*, Fig. S3*A*), activation function (e.g., Sigmoid in *SI Appendix*, Fig. S3*B*), and weight initialization (*SI Appendix*, Fig. S4). Heavy-tailed updates were also observed in a character-level language prediction task using a Transformer architecture (*SI Appendix*, Fig. S5). Moreover, heavy-tailed behavior was evident under full-batch training for SGD-family optimizers, where no mini-batch-induced noise was present (*SI Appendix*, Fig. S6), indicating that this phenomenon does not necessarily arise from mini-batch stochasticity. In all cases, the heavy-tailed nature of the update distributions remained evident. These results suggest that the emergence of heavy tails is not simply a consequence of specific model architectures, tasks, or training stages, but instead reflects a fundamental and intrinsic property of gradient-based learning dynamics.

### Heavy-Tailed Update Distributions Arise from Maximum Entropy Principle Under the Mutual Information Constraint.

The consistent emergence of heavy-tailed updates across diverse training conditions prompts a fundamental question: What underlying mechanism gives rise to this phenomenon? ANNs, as probabilistic systems trained for classification tasks, update their parameters to improve the likelihood of correctly activating target neurons (or units). While this is traditionally framed as loss minimization (34), it can also be viewed as a constrained probabilistic process.

From this perspective, learning seeks to maximize the entropy of the update distribution, H(p(Δw)), to retain flexibility in exploring the solution space, while ensuring task-relevant performance by constraining the mutual information between updates (i.e., Δw) and neuron activations (denoted by a), expressed as I(Δw; a). This trade-off between maximizing entropy and improving task-relevant information naturally leads to the emergence of heavy-tailed update distributions,

[2] $$\begin{array}{c} \max H\left(p\left(\Delta w\right)\right)\;\text{s.t.}I\left(\Delta w;\;a\right)=C_{I}, \end{array}$$

where $C_{I}$ is a constant representing the mutual information constraint. We applied the method of Lagrange multipliers, incorporating the mutual information constraint into the entropy maximization framework (see *SI Appendix*, section IV for a detailed derivation). This yields the following solution:

[3] $$\begin{array}{c} p\left(\Delta w\right)=e^{(-1-\mu -\lambda D_{\mathrm{KL}}\left(p\left(a|\Delta w\right)||p\left(a\right))\right)}, \end{array}$$

where μ corresponds to the normalization condition on p(Δw) and $D_{\text{KL}}$ denotes the Kullback–Leibler (KL) divergence between the conditional distribution p(a|Δw) and the marginal distribution p(a). Intuitively, this KL divergence quantifies the information gain about neural activity a induced by a specific update Δw. For small perturbations in parameter space, the KL divergence can be locally approximated via the Fisher information matrix (FIM) (35) as $D_{\mathrm{KL}}\left(p\left(a|\gamma \right)||p\left(a\right)\right)=\frac{1}{2}\gamma^{T}F\left(\Delta w\right)\gamma +{o(||\gamma ||}^{2})$, with γ=Δw, where the FIM quantifies the sensitivity of p(a|Δw) to changes in Δw, defined as $F_{\mathrm{kl}}\left(\Delta w\right)=E_{p(a|\Delta w)}\left[\frac{\partial \;\log\;p(a|\Delta w)}{\partial \;\Delta w_{k}}\frac{\partial \;\log\;p(a|\Delta w)}{\partial \;\Delta w_{l}}\right]$. Leveraging the minimal weight-activity duality (6), which describes how changes in activity can be minimally offset by adjusting incoming weights without altering preactivation or output, and assuming Gaussian mini-batch sampling noise (36, 37) (empirically validated in *SI Appendix*, Fig. S13), we derived the scaling behavior of the FIM:

[4] $$\begin{array}{c} F_{\mathrm{kl}}\left(\Delta w\right)=\frac{1}{{\left|\Delta w\right|}^{2}}G_{\mathrm{kl}}, \end{array}$$

where $G_{\mathrm{kl}}$ is a direction-dependent matrix independent of the magnitude |Δw| (*SI Appendix*, sections I and II).

**Fig. 3.**
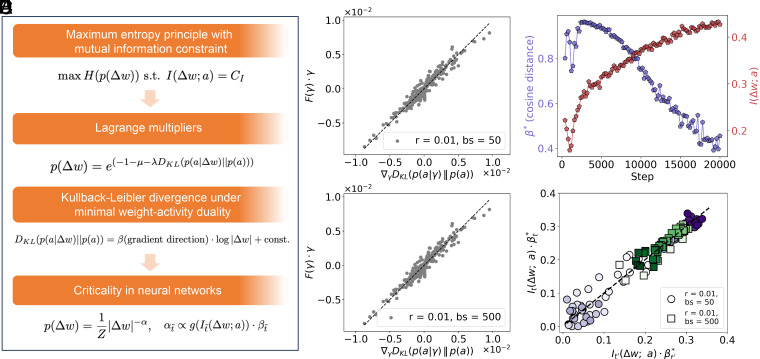
Theoretical derivation and empirical validation for the emergence of power-law-like update distributions. (*A*) Schematic overview of the theoretical framework. Starting from an entropy-maximization formulation constrained by mutual information, the resulting distribution of updates takes the form $p\left(\Delta w\right)\propto {\left|\Delta w\right|}^{-\alpha }$, where the exponent α depends on the mutual information I(Δw; a), with a denoting neuron (or unit) activations, and the gradient-direction factor β. Here, μ enforces the normalization condition ∫p(Δw)dΔw=1, while λ corresponds to the mutual information constraint. The derivation incorporates Lagrange multipliers and Kullback–Leibler (KL) divergence approximation. (*B* and *C*) Empirical validation of the local quadratic approximation $\nabla_{\gamma }D_{\mathrm{KL}}\left(p\left(a|\gamma \right)\;\|\;p\left(a\right)\right)\approx F\left(\gamma \right)\cdot \gamma$, where γ=Δw, under different batch sizes (bs=50 and bs=500) with fixed learning rate r=0.01. Dashed lines indicate the identity line for reference. (*D*) Evolution of β-proxy $\beta^{*}$ (approximated by the cosine distance between the leading principal components obtained via principal component analysis (PCA) on updates within adjacent training intervals; blue) and mutual information I(Δw; a) (red) over training steps, showing a clear transition in learning dynamics (bs=500, r=0.01). (*E*) Empirical proxy of the power-law exponent using the product $I_{t}\left(\Delta w;\;a\right)\cdot \beta_{t}^{*}$ across training steps. Each point represents a pairwise comparison between $I_{t}\cdot \beta_{t}^{*}$ and $I_{t^{\prime }}\cdot \beta_{t^{\prime }}^{*}$ with $t^{\prime }=t+2$, under different training configurations. A dashed identity line is shown. The alignment across both small and large batch sizes (bs=50 and bs=500, r=0.01) indicates that the product remains approximately invariant across nearby steps (from 0 to 20,000 steps). Marker color denotes training progress, with darker colors representing later steps. This stepwise proxy-based observation supports the finding that the power-law exponent remains stable during nonequilibrium learning.

To quantify the KL divergence $D_{\text{KL}}\left(p\left(a|\Delta w\right)\;\|\;p\left(a\right)\right)$, we adopted a path integral approach and expressed it as $\int_{\gamma }\nabla_{\Delta w^{\prime }}D_{\text{KL}}\left(p\left(a|\Delta w^{\prime }\right)\;\|\;p\left(a\right)\right)\cdot d\Delta w^{\prime },$ where γ denotes a continuous path in parameter space from 0 to Δw. Such a path-based formulation reflects the nonequilibrium nature of learning, akin to the Maximum Caliber framework (38, 39), in which information accumulates along dynamic paths in configuration space. By parameterizing this path as γ(t)=t·Δw for 0≤t≤1, and noting that $\frac{d\gamma (t)}{\mathrm{dt}}=\Delta w$, the integral simplifies to $\int_{0}^{1}\nabla_{\gamma (t)}D_{\text{KL}}\left(p\left(a|t\Delta w\right)\;\|\;p\left(a\right)\right)\cdot \Delta w\;dt$. To evaluate the integrand, we employed a local quadratic approximation of the KL divergence in the vicinity of Δw=0, expressed as $\nabla_{\gamma }D_{\text{KL}}\left(p\left(a|\gamma \right)\;\|\;p\left(a\right)\right)\approx F\left(\gamma \right)\cdot \gamma$. This approximation is supported by our numerical simulations (see Fig. 3 *B* and *C* and *SI Appendix*, Fig. S8 for different training steps). Applying the chain rule with γ(t)=tΔw, we obtained $\frac{d}{\mathrm{dt}}D_{\text{KL}}\left(p\left(a|t\Delta w\right)\;\|\;p\left(a\right)\right)={\left(t\Delta w\right)}^{T}\cdot F\left(t\Delta w\right)\cdot \Delta w$, where the transpose ensures a valid inner product. Substituting the FIM scaling relation (Eq. **4**), we simplified the derivative as $\frac{d}{\mathrm{dt}}D_{\text{KL}}=\frac{\Delta w^{T}G\Delta w}{{t|\Delta w|}^{2}}=\frac{g(\theta )}{t}$, where $g\left(\theta \right)=\frac{\Delta w^{T}G\Delta w}{{\left|\Delta w\right|}^{2}}$ is a scalar depending only on the direction $\hat{w}=\Delta w/\left|\Delta w\right|$. To regularize the divergence at t=0, we introduced a small cutoff $\epsilon =\frac{|\Delta w_{0}|}{\left|\Delta w\right|}$, yielding

[5] $$\begin{array}{c} D_{\mathrm{KL}}\left(p\left(a|\Delta w\right)||p\left(a\right)\right)=g\left(\theta \right)\int_{\epsilon }^{1}\frac{1}{t}dt=\beta \log\left|\Delta w\right|+\;\text{const.}, \end{array}$$

where β=g(θ) denotes a direction-dependent constant parameter that may vary during training (*SI Appendix*, section III). Combining Eqs. **3** and **5** (Fig. 3*A*), we obtained the final expression for the distribution over updates:

[6] $$\begin{array}{c} p\left(\Delta w\right)=\frac{1}{Z}{\left|\Delta w\right|}^{-\alpha }, \end{array}$$

where α=λβ and the normalization constant is given by $Z=e^{\left(1+\mu +\lambda C_{w}\right)}$, where $C_{w}$ is a direction-dependent constant determined by the reference scale (*SI Appendix*, section IV). The exponent α is thus determined by both the strength of the mutual information constraint (represented by the Lagrange multiplier λ) and the directional sensitivity β, which reflects the local geometry of the parameter space. Since λ enforces the constraint on mutual information, it acts as a monotonic function of the mutual information, i.e., $\lambda =g\left(I(\Delta w;\;a)\right)$, where g(·) is a monotonic increasing function.

### Validation of the Information-Driven Self-Organization Learning Framework.

To empirically validate our theoretical framework, we analyzed the evolution of mutual information and gradient directionality throughout training. While the value of the exponent α cannot be directly estimated, owing to the complexity of the Lagrange multiplier’s functional form g(·) and the variability of the full-parameter gradients, we instead tracked its relative changes during training through empirically measurable proxy quantities. Specifically, we extracted updates from one to five fully connected layers across training steps ranging from initialization to 20,000, using SGD with a fixed learning rate r. At each training step, the updates were concatenated into a high-dimensional vector, forming a trajectory in parameter space. To quantify the local consistency of gradient directions, corresponding to the exponent β, we partitioned training steps into consecutive nonoverlapping intervals (e.g., every 200 steps) and performed PCA on the updates within each interval. We then computed the cosine distance between the leading principal components of adjacent intervals (denoted as $\beta^{*}$), providing a coarse-grained measure of how the full-scale gradient direction evolves over time. To estimate the mutual information, associated with λ, we computed the joint and marginal distributions between the updates and the corresponding unit activations a using histogram-based binning (see *Materials and Methods* for mutual information estimation and *SI Appendix*, Fig. S9). These proxy-based estimations allow us to track the dynamics of task-relevant information during training and relate them to the observed heavy-tailed updates.

Fig. 3*D* illustrates an inverse trend between mutual information and gradient directionality during training: while I(Δw; a) (associated with λ) increased steadily during training, $\beta^{*}$ gradually declined with fluctuations. To evaluate the stability of the heavy-tailedness of power-law-like distributions α=λβ, we computed the product $I_{t}\left(\Delta w;\;a\right)\cdot \beta_{t}^{*}$ at training step t, and compared it with $I_{t^{\prime }}\left(\Delta w;\;a\right)\cdot \beta_{t^{\prime }}^{*}$ at a nearby step $t^{\prime }=t+2$. The strong Spearman correlations (0.94 for bs=50 and 0.91 for bs=500; Fig. 3*E*) indicate that the product remains approximately invariant across adjacent steps, i.e., $I_{t^{\prime }}\left(\Delta w;\;a\right)\cdot \beta_{t^{\prime }}^{*}\approx I_{t}\left(\Delta w;\;a\right)\cdot \beta_{t}^{*}$. Similar results were observed for $t^{\prime }=t+1$ (*SI Appendix*, Fig. S10). A two-sided one-sample t test on the difference ($I_{t^{\prime }}\cdot \beta_{t^{\prime }}^{*}-I_{t}\cdot \beta_{t}^{*}$) found no statistically significant deviation from zero across all training configurations (P>0.05; see *SI Appendix*, Fig. S11). The color-coded progression across training steps displays a range-limited fluctuating pattern (Fig. 3*E* and *SI Appendix*, Fig. S10). Such range-constrained variation provides empirical support for our theoretical framework, suggesting that the observed heavy-tailedness in updates arises from a dynamic yet regulated learning process shaped by entropy maximization and mutual information constraint.

### Multiscale Ruggedness in the Loss Landscape at Local Minima.

While our theoretical and empirical analyses show that update distributions exhibit heavy-tailed behavior during nonequilibrium learning, the presence of stochastic gradient noise complicates the interpretation of the underlying geometric structure. On one hand, mini-batch noise facilitates exploration and may help the model reach minima associated with better generalization (40). On the other hand, it introduces fluctuations that obscure the intrinsic features of the loss landscape itself (41). To isolate these noise-induced effects from the true geometric landscape, we conducted controlled perturbation experiments on well-trained models after convergence, in the absence of stochastic mini-batch sampling.

**Fig. 4.**
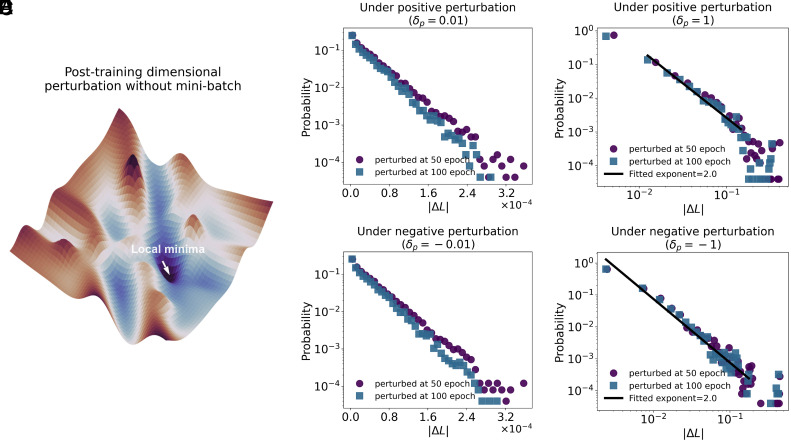
Posttraining perturbation analysis reveals scale-dependent loss landscape geometry. (*A*) Illustration of posttraining dimensional perturbation evaluated without mini-batch sampling. Loss is computed over the entire training set to eliminate stochastic noise. (*B* and *C*) Probability of loss change |ΔL| under small perturbations ($\delta_{p}=\pm 0.01$) at two different training checkpoints (50 and 100 epochs), plotted in semi-log scale (x-axis in linear scale with scientific notation and y-axis in log scale). Both positive and negative perturbations produce exponential-like decay, indicating smooth geometry near local minima. (*D* and *E*) Probability distributions under large perturbations ($\delta_{p}=\pm 1$) show heavy-tailed scaling behavior. Power-law fits yield exponents $\alpha_{L}\approx 2.0$, demonstrating that perturbations beyond the local basin exhibit scale-invariant ruggedness in the loss landscape.

Specifically, after training the models to convergence (e.g., 50 or 100 epochs, with each epoch consisting of $N_{t}=N_{s}/B_{s}$ training steps, where $N_{s}$ denotes training set size and $B_{s}$ is batch size), we randomly selected 5,000 parameters per layer across all five fully connected layers, resulting in a total of 25,000 perturbation trials. For each selected dimension $w_{i}$, we applied symmetric perturbations of the form $w_{i}\to w_{i}+\delta_{p}$ with $\delta_{p}=\pm 0.01$ for small perturbations and $\delta_{p}=\pm 1$ for large perturbations. These values were chosen based on the empirical parameter ranges (*SI Appendix*, Fig. S15). To eliminate sampling-induced noise, all loss evaluations L(·) were performed on the full training dataset. The perturbation response is defined as the absolute loss change $\Delta L=|L\left(w_{i}+\delta_{p}\right)-L\left(w_{i}\right)|$. Our result shows a scale-dependent geometry in the loss landscape: the distribution of ΔL follows an exponential decay under small perturbations, indicating smooth curvature in local basins (Fig. 4 *B* and *C*). However, for larger perturbations, the distribution transitions to a power-law form, $p\left(\Delta L\right)\propto {\left|\Delta L\right|}^{-\alpha_{L}}$, with exponent $\alpha_{L}\approx 2$, consistent across both positive and negative perturbations (Fig. 4 *D* and *E*). This exponential-to-power-law transition highlights a multiscale ruggedness (*SI Appendix*, Fig. S14), where the landscape appears locally flat but exhibits scale-free properties at larger scales.

To ensure that the perturbation-based analysis reflects the intrinsic geometry of the loss landscape (42), we validated it using synthetic loss surfaces with predefined curvature including Gaussian, power-law, and exponential forms. The resulting perturbation-induced loss responses closely matched the expected statistical patterns (*SI Appendix*, Figs. S16–S18), supporting the validity of our approach. These results suggest that SGD favors regions of parameter space where locally flat minima are embedded within a globally rugged landscape. The presence of multiscale ruggedness provides a geometric basis for the effectiveness of SGD in both optimization and generalization.

### Scaling Behavior in the Timing of Large Updates.

Inspired by natural phenomena such as earthquakes where both event magnitudes and interevent times follow power-law distributions (43, 44), we investigated whether similar temporal scaling behavior arises during neural network training. Specifically, we examined the distribution of time intervals between consecutive large-magnitude updates. The magnitude of updates |Δw| exhibits heavy-tailed distributions (Fig. 2). To characterize temporal dynamics, we introduced a threshold $V_{\theta }$ and identified large updates as those exceeding the top 0.5% or 1% of all observed magnitudes. We recorded the training steps ${\left\{t_{k}\right\}}_{k=1}^{K}$ at which these large updates occurred that satisfy $|\Delta w_{i}^{\left(t_{k}\right)}|>V_{\theta }$. The corresponding interevent times are defined as $\Delta t_{k}=t_{k+1}-t_{k}$ for k=1,2,…,K−1, where K is the total number of large-update events.

We found that the distribution of interevent time intervals also follows a heavy-tailed pattern, with fitted power-law exponents of approximately 2.7 for MLPs and 2.5 for CNNs. These exponent values are comparable to those reported in diverse scale-invariant systems, including seismic activity (44), financial volatility (45), and neuronal avalanches (46). The emergence of temporal scaling behavior suggests that large updates are temporally clustered rather than occurring uniformly over time, revealing an underlying nontrivial organization in gradient-based learning. Thus, temporal scaling law reinforces the interpretation of neural network training as a self-organizing, nonstationary process governed by complex and scale-free dynamics.

## Discussion

Learning dynamics in neural networks indicate a form of self-organization governed by information-driven constraints. We found that the mutual information I(Δw; a) between parameter updates and activations increases and gradually approaches a saturation point in training (Fig. 3 and *SI Appendix*, Fig. S9). In parallel, the entropy H(p(Δw)) of update distributions increased initially but later stabilizes (*SI Appendix*, Fig. S12). We observed a competing relationship between mutual information and entropy (*SI Appendix*, Fig. S12), consistent with our theoretical formulation: mutual information constrains updates to be informative, while entropy reflects the degree of permissible variability. Their interplay suggests a dynamic balance between random exploration and task-relevant adaptation, which is a feature of systems evolving far from equilibrium.

Since our theoretical framework is grounded in first principles, specifically derived from an unbiased evolution under informational constraints, it is conceptually aligned with the Maximum Caliber framework (39) from nonequilibrium statistical mechanics, which extends entropy maximization to entire trajectories. Rather than modeling full learning paths, we focus on local update statistics and show that heavy-tailed distributions arise from the maximum entropy principle under a mutual information constraint.

A key observation is that heavy-tailed update distributions consistently appear across a range of neural network architectures (MLP, CNN, and Transformer, see Fig. 2 and *SI Appendix*, Fig. S5) and training regimes (rapid loss decrease and steady convergence). The flattening of the far tail (Fig. 2 and *SI Appendix*, Figs. S2–S5) reflects a statistical sampling effect caused by the rarity of large updates. Previous studies have observed heavy tails in mini-batch-induced gradient noise (47, 48) and in multiplicative-noise-driven stationary distribution of weights (49). While mini-batch sampling introduces stochasticity into the optimization process, our results suggest that it does not have to be the only one source of noise facilitating exploration. Likewise, multiplicative noise is not the main factor, as it cannot account for the persistent heavy-tailed behavior observed across different training stages. Specifically, we examine the empirical distributions of parameter updates and find that the heavy-tailed update distributions persist across training tasks and stages, and also arise under full-batch training across different SGD-family optimizers, where no mini-batch-induced noise is present (*SI Appendix*, Fig. S6). This numerical observation is consistent with the theoretical scaling $F\propto {\left|\Delta w\right|}^{-2}$ (Eq. **4**), which arises from the information-entropy trade-off, rather than from a specific source of noise. To explain why such heavy-tailed phenomena exist across different cases, we propose a more fundamental origin: heavy-tailed update distributions arise intrinsically from information-driven self-organization rooted in the optimization process (i.e., the search for an effective solution).

While our theoretical derivation shows that power-law distributions naturally arise from entropy maximization under the mutual information constraint, empirical update distributions may exhibit deviations from exact power-law forms due to both structural and functional constraints. Specifically, learning rate and activation saturation act as functional constraints that restrict the gradient range during training (*SI Appendix*, Figs. S2 and S3*B*), whereas purely linear networks (without activation; *SI Appendix*, Fig. S3*C*) exhibit spectral concentration in gradient propagation by repeated multiplication, with more frequent small updates. Moreover, Xavier initialization slightly underestimates the variance required for ReLU activations, introducing a structural constraint at initialization that produces an early saturation of updates during later training stages (*SI Appendix*, Fig. S4). Therefore, although many of these distributions exhibit power-law-like behavior, we do not exclude the possibility that other heavy-tailed forms may also provide a good fit. Since the log-normal family asymptotically includes power-law behavior in finite ranges (28, 50), we interpret neural learning dynamics as displaying scale-free behavior governed by heavy-tailed distributions.

**Fig. 5.**
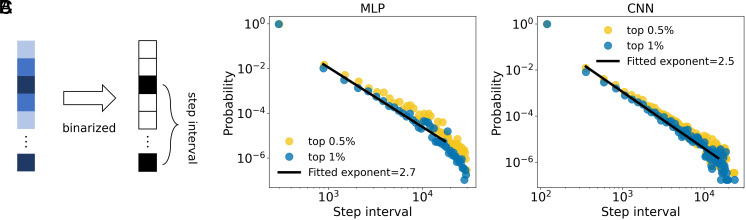
Scaling behavior in the timing of large updates. (*A*) Graphical depiction of the procedure for extracting step intervals between large updates. The magnitudes of updates |Δw| are first thresholded to identify large events (top 0.5% or 1%) within training period (steps 0 to 30,000) and then binarized. From this, we compute the interevent step intervals between successive large updates. (*B* and *C*) Distributions of interevent time intervals for MLP (∼10^5^ parameters, *B*) and CNN (∼10^4^ parameters, *C*) models show power-law-like behavior. Each solid line shows a least-squares fit applied to the data points within its corresponding range. The fitted exponents are approximately 2.7 for MLP and 2.5 for CNN, respectively. These results indicate that large updates are not randomly distributed in time, but are temporally clustered in a scale-invariant fashion.

To examine the underlying landscape in the absence of stochastic noise, we performed a perturbation-based analysis, which showed multiscale ruggedness: locally flat basins with exponential decay under small perturbations gradually transitioning to power-law-like ruggedness at larger scales (Fig. 4 and *SI Appendix*, Fig. S14). If a model is trained with deterministic gradient descent (GD) that closely follows the landscape, it tends to become trapped at saddle points or sharp minima, resulting in poor generalization performance (40). This occurs because GD focuses solely on the mutual information constraint ignoring exploration. In contrast, SGD introduces mini-batch noise that enables the model to explore broader regions of the landscape, thereby favoring flatter minima and improving generalization (40). Mini-batch sampling can prompt exploration, as can the learning rate. Our result shows that even under full-batch training, SGD with a moderate learning rate (neither too small nor too large) achieves good performance, characterized by heavy-tailed update distribution (*SI Appendix*, Fig. S6*A*). This power-law-like behavior directly reflects the balance between exploration (i.e., maximum entropy) and task relevance (i.e., the mutual information constraint). If the learning rate is too small, the learning process mainly focuses on task relevance locally and becomes trapped, while if it is too large, the process is dominated by exploration and becomes unstable. Therefore, for the model to perform well, it needs to strike a trade-off between entropy and mutual information, balancing exploration, and task relevance. Such a balance explains why heavy-tailed update distributions can persist and are essential for achieving effective solutions under scale-dependent landscapes. Beyond distributional properties, we also uncovered temporal signatures of criticality (Fig. 5). The intervals between large update events follow heavy-tailed distributions, resembling event timing in critical systems such as earthquakes or neuronal avalanches.

There are several directions worth exploring in future work. Our current framework is developed for SGD, whose update direction follows the log-likelihood gradient, a property leveraged in the path-integral formulation of the KL divergence approximation (*SI Appendix*, section III). This property holds exactly when the loss corresponds to the negative log-likelihood (e.g., cross-entropy) and holds approximately for MSE under a Gaussian noise assumption. However, adaptive methods such as Adam can deviate from the log-likelihood gradient due to per-parameter rescaling, which also effectively introduces an algorithmic cutoff that limits the learning step size (*SI Appendix*, Fig. S7*A*). One possible reason why SGD tends to generalize better than adaptive methods (51) is that SGD preserves power-law-like update statistics through a self-organizing process that balances entropy and mutual information, whereas adaptive methods bias this balance by preventing large exploratory updates (*SI Appendix*, Fig. S7). Extending the analysis to adaptive optimizers may provide a more refined understanding of how different update rules influence the information-entropy trade-off. Furthermore, incorporating time-resolved trajectory modeling (19) could deepen our understanding of how local update statistics give rise to global learning behavior.

In summary, we present both theoretical and numerical evidence that learning in neural networks exhibits information-driven self-organization, characterized by heavy-tailed updates and temporally clustered dynamics. These features parallel behaviors in nonequilibrium physical systems and suggest that the emergent properties of learning can be understood through the lens of statistical physics. This perspective not only enhances our understanding of emergent dynamics in neural networks but also opens directions for developing learning algorithms grounded in principles from statistical mechanics (52, 53).

## Materials and Methods

### Dataset Information.

In this study, we used two benchmark image datasets: MNIST and CIFAR-10, both of which consist of 10 distinct classes. The MNIST dataset contains grayscale images of handwritten digits ranging from 0 to 9, with each image sized at 28×28 pixels. It includes 60,000 training samples and 10,000 test samples. In contrast, the CIFAR-10 dataset comprises 60,000 color images of size 32×32 pixels across 10 object classes, with 6,000 images per class. The training-to-test split is 50,000 to 10,000. The classes include airplane, automobile, bird, cat, deer, dog, frog, horse, ship, and truck.

### Neural Network Architectures.

We evaluated multiple widely used feedforward architectures: multilayer perceptrons (MLPs) and convolutional neural networks (CNNs), chosen to cover different model regimes. Similar results were obtained using a Transformer-based architecture, as reported in *SI Appendix*. Specifically, we varied the batch size from 4 to 1,000, the initialization scheme [Kaiming uniform initialization (54) and Xavier (Glorot) normal initialization (55)], activation functions (ReLU and Sigmoid), and the learning rate from 0.1 to 0.01 across configurations.

#### MLP for MNIST.

We constructed three variants of fully connected MLPs trained on the MNIST dataset, each differing in width and depth to span different model capacities: The small-scale MLP contains approximately $10^{4}$ trainable parameters, comprising four hidden layers with 50 units each and a final output layer with 10 units, totaling five layers. The medium-scale MLP has around $10^{5}$ parameters with one hidden layer of 128 units. The large-scale MLP reaches $10^{6}$ parameters with four hidden layers of 1000 units each. All MLPs are applied ReLU activation after each hidden layer and a final softmax output layer with 10 units for digit classification.

#### CNN for CIFAR-10.

We used a CNN trained on the CIFAR-10 dataset, roughly $10^{4}$ parameters. This model includes two convolutional layers (32 and 64 channels), each followed by ReLU activation, batch normalization, and max pooling. The resulting features are flattened and passed through two fully connected layers with 512 and 10 units, respectively. The CNN is tailored for spatial feature extraction in color images and offers a structurally distinct comparison to MLP.

### Initialization Schemes.

Unless otherwise specified, all networks are initialized using the Kaiming uniform initialization method (54), which is designed for ReLU-based neural networks to preserve signal variance through depth. For a layer with $f_{\text{in}}$ input units, the Kaiming uniform initialization samples weights w from the interval:

$$w∼U\left(-\sqrt{\frac{6}{f_{\text{in}}}},\sqrt{\frac{6}{f_{\text{in}}}}\right).$$

To examine the role of initialization on learning dynamics, we also evaluated the Xavier (Glorot) normal initialization (55). In this case, weights are drawn from a zero-mean Gaussian distribution with variance:

$$w∼N\left(0,\frac{2}{f_{\text{in}}+f_{\text{out}}}\right),$$

where $f_{\text{in}}$ and $f_{\text{out}}$ denote the number of input and output units of the layer, respectively. These initializations aim to balance the flow of forward and backward signals at the start of training and were found not to qualitatively influence the heavy-tailed update distributions, as shown in *SI Appendix*, Fig. S4.

### Stochastic Gradient Descent.

We employed stochastic gradient descent (SGD) as the optimization algorithm for training all neural network models in this study. SGD updates model parameters iteratively by computing the gradient of the loss function with respect to a randomly sampled mini-batch of data. At each iteration t, the parameters $w_{t}$ are updated according to:

$$w_{t+1}=w_{t}-r\nabla_{w}L\left(w_{t};\;B_{t}\right),$$

where r denotes the learning rate, L is the loss function (e.g., cross-entropy or mean squared error), and $B_{t}$ is the mini-batch of training data sampled at step t. In this study, we fixed the mini-batch size to 64 unless stated otherwise. To isolate the intrinsic properties of updates, we did not employ any additional regularization techniques such as weight decay or dropout.

### Loss Functions.

We considered two commonly used objective functions in neural network training: cross-entropy loss and mean squared error (MSE) loss. The cross-entropy loss is typically used for classification tasks and is defined as

$$L_{\text{CE}}=-\sum_{i=1}^{N_{C}}y_{i}\log\left({\hat{y}}_{i}\right),$$

where $N_{C}$ is the number of classes, $y_{i}$ is the one-hot encoded ground truth label, and ${\hat{y}}_{i}$ is the predicted probability for class i. This cross-entropy loss encourages the predicted distribution to match the target distribution and penalizes incorrect confidence. For comparison, we also employed MSE loss, which is typically used for regression tasks or continuous-valued outputs,

$$L_{\text{MSE}}=\frac{1}{N_{d}}\sum_{i=1}^{N_{d}}{\left(y_{i}-{\hat{y}}_{i}\right)}^{2},$$

where $N_{d}$ is the number of output dimensions, and $y_{i}$ and ${\hat{y}}_{i}$ are the ground truth and predicted values, respectively. This loss measures the average squared difference between predicted and true values and is sensitive to large deviations.

### Activation Functions.

Activation functions introduce nonlinearity into neural networks, enabling them to model complex and nonlinear relationships. In our study, we employed two activation functions: the Rectified Linear Unit (ReLU) and the Sigmoid function. The ReLU activation is defined as

ReLU(x)= max(0,x),

which preserves positive inputs and zeroes out negative values, facilitating sparse representations and mitigating the vanishing gradient problem. In contrast, the Sigmoid activation maps real-valued inputs to the interval [0,1] and is defined as

$$\text{Sigmoid}\left(x\right)=\frac{1}{1+e^{-x}},$$

which is often used in binary classification and probabilistic interpretation tasks.

### Mutual Information Estimation.

To quantify the mutual information between model activations a and updates |Δw|, we estimated the joint and marginal distributions using histogram-based binning. Here, ΔW denotes the concatenation (or stacking) of updates Δw across layers, resulting in a high-dimensional representation at a given training step. Similarly, A denotes the corresponding concatenated neuron/unit activations (*SI Appendix*, Fig. S9) in response to inputs. To ensure comparability and numerical stability, both ΔW and A are standardized while preserving the relative magnitudes across entries:

$$\tilde{\Delta W}=\frac{\Delta W-E[\Delta W]}{\sigma (\Delta W)},\;\tilde{A}=\frac{A-E[A]}{\sigma (A)}.$$

We then constructed a 2D histogram $H_{\mathrm{ij}}$ with 50×50 bins to approximate the joint distribution $P({\tilde{\Delta w}}_{i},{\tilde{a}}_{j})$, where ${\tilde{\Delta w}}_{i}$ and ${\tilde{a}}_{j}$ represent entries from the concatenated vectors $\tilde{\Delta W}$ and $\tilde{A}$, respectively, and normalized it to obtain a valid probability distribution:

$$P\left({\tilde{\Delta w}}_{i},{\tilde{a}}_{j}\right)=\frac{H_{\mathrm{ij}}}{\sum_{i,j}H_{\mathrm{ij}}}.$$

The marginal distributions are computed by summing over respective dimensions:

$$P\left({\tilde{\Delta w}}_{i}\right)=\sum_{j}P\left({\tilde{\Delta w}}_{i},{\tilde{a}}_{j}\right),\;P\left({\tilde{a}}_{j}\right)=\sum_{i}P\left({\tilde{\Delta w}}_{i},{\tilde{a}}_{j}\right).$$

To prevent numerical instability caused by zero-probability entries, a small constant $10^{-10}$ was added to all probabilities before computing the mutual information:

$$I\left(\Delta w;\;a\right)=\sum_{i,j}P\left({\tilde{\Delta w}}_{i},{\tilde{a}}_{j}\right)\log\left(\frac{P({\tilde{\Delta w}}_{i},{\tilde{a}}_{j})}{P\left({\tilde{\Delta w}}_{i}\right)\;P\left({\tilde{a}}_{j}\right)}\right).$$

This estimation quantifies the amount of information shared between model activations and the magnitude of updates.

### Gradient Direction Estimation.

To assess how gradient directions evolve during training, we tracked proxy directions of parameter updates across steps. At each training step, we concatenated the updates from all fully connected layers to a high-dimensional vector representing the overall parameter update. To quantify directional changes, we grouped the training steps into nonoverlapping intervals (e.g., every 200 steps), and performed principal component analysis (PCA) within each interval to identify the dominant update direction. We then computed the cosine distance between the leading principal components of adjacent intervals to measure how the main update direction shifts over training. PCA serves two purposes in this section: 1) it reduces step-to-step noise by consolidating multiple updates into a dominant trend, and 2) it provides a distilled summary of directional dynamics in high-dimensional parameter spaces, where raw update vectors may be sparse or noisy. This approach captures coarse-grained transitions in gradient directions throughout training.

### Simulated Synthetic Landscapes with Predefined Ruggedness.

To validate our perturbation analysis to different levels of ruggedness in the loss landscape, we constructed high-dimensional synthetic loss surfaces that mimic diverse curvature profiles. Each synthetic landscape is modeled as a weighted paraboloid of the form:

$$f\left(x\right)=\sum_{i=1}^{n}c_{i}x_{i}^{2},$$

where $x\in R^{n}$ is a randomly initialized input vector and $c_{i}$ denotes the curvature coefficient along dimension i. The vector x is sampled from a uniform distribution over ${\left[0,1\right]}^{n}$, and the curvature coefficients ${\left\{c_{i}\right\}}_{i=1}^{n}$ are drawn from one of several distributions to simulate varying degrees of landscape ruggedness, including Gaussian, power-law, exponential, and lognormal distribution. We perturbed each coordinate $x_{i}$ of x by adding or subtracting a fixed scalar $\delta_{p}$ and measured the resulting change in the function output:

$$\Delta f_{i}=\left|f\left(x_{\text{perturbed}}\right)-f\left(x\right)\right|.$$

This process was repeated for each dimension i. The dimensionality was fixed at n=50,000 to emulate the high dimensionality of neural network parameter spaces. The resulting $\Delta f_{i}$ distributions were stored for subsequent statistical analysis of landscape ruggedness. Unlike in the ANN setting, where the loss landscape is implicitly shaped by the model architecture, data, and optimization trajectory and can only be probed by perturbing the model parameters $w_{i}$, our synthetic formulation explicitly separates the roles of geometry and probing. In particular, the curvature coefficients $c_{i}$ define the underlying geometric profile of the landscape, while perturbations are applied to the input coordinates $x_{i}$. This separation-based synthetic landscape allows us to benchmark our perturbation-based approach in detecting known ruggedness profiles and provides a testbed to assess whether the measured $\Delta f_{i}$ statistics (e.g., exponential or power-law tails) truly reflect geometric ruggedness.

### Data Fitting.

We employed two complementary fitting approaches depending on the nature of the data and the goal of the visualization. For Figs. 1–3, we provided indicative reference dashed lines representing power-law-like behavior, intended to guide visual interpretation rather than reflect precise parameter estimates. In contrast, for Figs. 4 and 5, we fitted the empirical data points using quantitative regression. Specifically, power-law relationships were fitted using least-squares regression on log–log transformed data. Each fit was restricted to the range of data points aligned with the corresponding solid line to avoid low-signal or cutoff regions that could bias the slope estimate. For the analysis of mini-batch gradient distributions (*SI Appendix*, Fig. S13), we applied maximum likelihood estimation to fit Gaussian distributions. This fitting was implemented using scipy.stats.norm from the SciPy library (56), which provides statistically efficient estimates of the mean and SD under the assumption of normality.

## Supplementary Material

Appendix 01 (PDF)

## Data Availability

The source data and code used in this paper are openly available at GitHub https://github.com/xinyacheung/Heavytailed_updates_ANN (57).
