# Supplementary material for: Heavy-tailed update distributions arise from information-driven self-organization in nonequilibrium learning
Source: Proc Natl Acad Sci U S A. 2025 Dec 18;122(51):e2523012122. doi: 10.1073/pnas.2523012122 (PMC12745802; doi:10.1073/pnas.2523012122)
Supplement: Supplementary file 1 — Appendix 01 (PDF) [file pnas.2523012122.sapp.pdf]

Supporting Information for

Heavy-tailed update distributions arise from information-driven self-organization in non-equilibrium learning

Xin-Ya Zhang and Chao Tang

Chao Tang
E-mail: tangchao@westlake.edu.cn

This PDF file includes:

Supporting text
Figs. S1 to S18
Tables S1 to S2
SI References

Supporting Information Text

I. Conditional distribution of activity given weight update

A. Weight-Activity duality. The weight-activity (W–A) duality in artificial neural networks characterizes how a change in the activity of a hidden layer can be compensated by a minimal change in the incoming weights, such that the pre-activation and hence the network output remains unchanged. In the context of feed-forward networks, the weight update rule with minimal W-A duality is given by (1):

$$\Delta w_{ij} = \sum_{i'} \frac{a_i \Delta a_{i'}}{\|a\|^2} w_{i'j}, \tag{1}$$

where w_{ij} represents the weight connecting neuron i to neuron j, a_i denotes the activity of neuron i, $\Delta a_{i'}$ is the change in the activity of neuron i' and $\|a\|^2 = \sum_i a_i^2$ is the squared norm of the activity vector.

We now derive this minimal update rule as follows (see also (1) for additional details). Consider two consecutive fully connected layers $(s-1)$ and s, where the pre-activation of neuron j in layer s is:

$$z_j^{[s]} = \sum_i w_{ij} a_i^{[s-1]}, \tag{2}$$

with $a_i^{[s-1]}$ the activation from the previous layer. To preserve the activation $a_j^{[s]} = \phi(z_j^{[s]})$ after changing activity in layer $(s-1)$, we require the pre-activation to remain unchanged:

$$z_j^{[s]} = \sum_i w_{ij}^* a_i' = \sum_i w_{ij} a_i. \tag{3}$$

Subtracting Eq. 3 gives the linear constraint:

$$\sum_i (\Delta w_{ij} a_i + w_{ij} \Delta a_i) = 0, \tag{4}$$

where $\Delta w_{ij} = w_{ij}^* - w_{ij}$ and $\Delta a_i = a_i' - a_i$. We seek the minimal norm solution $\mathbf{w}^*$ satisfying the constraint, i.e., minimize:

$$\mathcal{L}(\Delta w, \varphi) = \sum_{i,j} (\Delta w_{ij})^2 + \sum_j \varphi_j \left(\sum_i (\Delta w_{ij} a_i + w_{ij} \Delta a_i) \right), \tag{5}$$

where φ_j is the Lagrange multiplier enforcing the constraint for neuron j. Taking the derivative of $\mathcal{L}$ with respect to Δw_{ij} gives:

$$\frac{\partial \mathcal{L}}{\partial \Delta w_{ij}} = 2\Delta w_{ij} + \varphi_j a_i = 0 \Rightarrow \Delta w_{ij} = -\frac{1}{2} \varphi_j a_i. \tag{6}$$

If we plug Eq. 4, we can solve for φ_j:

$$\sum_{i'} \left(-\frac{1}{2} \varphi_j a_{i'}^2 \right) + \sum_{i'} w_{i'j} \Delta a_{i'} = 0 \Rightarrow \varphi_j = -\frac{2 \sum_{i'} w_{i'j} \Delta a_{i'}}{\sum_i a_{i'}^2}. \tag{7}$$

Substituting Eq. 7 into Eq. 6 gives the W-A duality relationship as shown in Eq. 1. To account for noise sources such as mini-batch-induced noise, we incorporate it into the W-A duality relationship:

$$\Delta w_{ij} = \sum_{i'} \frac{a_i \Delta a_{i'}}{\|a\|^2} w_{i'j} + \varepsilon_{ij}, \tag{8}$$

where we assume that the noise terms ε_{ij} follow a Gaussian distribution $\varepsilon_{ij} \sim \mathcal{N}(0, \sigma^2)$, which is supported by previous studies (2, 3) and validated in Fig. S13.

B. Bayesian posterior conditional probability. Given activity a and activity changes Δa, the conditional probability of weight update Δw is:

$$p(\Delta w | a, \Delta a) = \frac{1}{K} e^{\left(-\frac{1}{2\sigma^2} \left\| \Delta w_{ij} - \sum_{i'} \frac{a_i \Delta a_{i'}}{\|a\|^2} w_{i'j} \right\|^2 \right)}, \tag{9}$$

where $K = (2\pi\sigma^2)^{n/2}$ is the normalization constant and n is the total number of weight parameters. Here we apply Bayes' theorem:

$$p(a, \Delta a | \Delta w) = \frac{p(\Delta w | a, \Delta a) \cdot p(a, \Delta a)}{p(\Delta w)}, \tag{10}$$

and substitute the forward conditional probability (Eq. 9) and the posterior probability is:

$$p(a, \Delta a|\Delta w) = \frac{1}{K(\Delta w)} e^{\left(-\frac{1}{2\sigma^2}\left\|\Delta w_{ij} - \sum_{i'} \frac{a_i \Delta a_{i'}}{\|a\|^2} w_{i'j}\right\|^2\right)} \cdot p(a, \Delta a), \tag{11}$$

where $K(\Delta w)$ is the normalization constant for the posterior probability in Bayesian inference, given the weight update Δw. To facilitate further analysis of the mutual information between weight update and activity, we next derive the conditional distribution of activity given the weight update:

$$p(a|\Delta w) = \int p(a, \Delta a|\Delta w)\, d(\Delta a). \tag{12}$$

This integral is generally intractable in closed form but can be approximated using the Laplace approximation. For given a and Δw, there exists an optimal Δa^* that maximizes the posterior probability $p(a, \Delta a|\Delta w)$:

$$\Delta a^* = \arg\max_{\Delta a} p(a, \Delta a|\Delta w). \tag{13}$$

Accordingly, we approximate the conditional distribution $p(a|\Delta w)$ by evaluating the posterior distribution at the optimal Δa^*:

$$p(a|\Delta w) \approx p(a, \Delta a^*|\Delta w). \tag{14}$$

If substituting the form of the joint posterior (Eq. 11), and assuming that the prior term $p(a, \Delta a^*)$ is weakly dependent on Δw, we obtain:

$$p(a|\Delta w) \propto e^{\left(-\frac{1}{2\sigma^2}\left\|\Delta w_{ij} - \sum_{i'} \frac{a_i \Delta a^*_{i'}}{\|a\|^2} w_{i'j}\right\|^2\right)}, \tag{15}$$

which is the expression used in subsequent derivations. Note that in the local neighborhood of Δw, both the normalization term $K(\Delta w)$ and the prior term $p(a, \Delta a^*)$ are treated as approximately constant. This approximation, commonly used in Bayesian and information-theoretic analyses, assumes that these terms vary slowly compared to the sharper variations of the likelihood, and thus can be absorbed into the proportionality (4–6).

II. Fisher information matrix

The Fisher Information Matrix (FIM) quantifies the local geometric structure of the parameter space in statistical models. In the context of neural networks, FIM characterizes how sensitively the distribution of activity depends on weight update. Formally, FIM is defined as:

$$F_{kl}(\Delta w) = \mathbb{E}_{p(a|\Delta w)}\left[\frac{\partial \log p(a|\Delta w)}{\partial \Delta w_k} \frac{\partial \log p(a|\Delta w)}{\partial \Delta w_l}\right]. \tag{16}$$

Here, Δw_k and Δw_l denote individual components of the weight update vector Δw. For a neural network with n weight parameters, the indices k and l each range from 1 to n, yielding in an $n \times n$ Matrix. FIM captures the curvature of the log-likelihood function around the current parameter values. Each entry F_{kl} measures how changes in parameters Δw_k and Δw_l jointly affect the log-likelihood of observing activity a.

A. Score function. FIM depends on the score function (i.e., the partial derivative $\frac{\partial \log p(a|\Delta w)}{\partial \Delta w_k}$). To compute the score function, we first note that it is the gradient of the log-likelihood with respect to the weight update, Δw:

$$\nabla_{\Delta w} \log p(a|\Delta w) = \frac{\partial \log p(a|\Delta w)}{\partial \Delta w}. \tag{17}$$

Using the Laplace approximation, we approximate the posterior distribution $p(a|\Delta w)$ by the joint distribution $p(a, \Delta a^*|\Delta w)$, i.e., Eqs. 14 and 15, where Δa^* is the optimal activity update that maximizes the posterior. To simplify notation, we define $\mu_{ij} := \sum_{i'} \frac{a_i \Delta a^*_{i'}}{\|a\|^2} w_{i'j}$. The log-likelihood then becomes:

$$\log p(a|\Delta w) = -\frac{1}{2\sigma^2} \|\Delta w_{ij} - \mu_{ij}\|^2 + C', \tag{18}$$

where C' is the constant. Differentiating with respect to Δw_{ij}, we obtain the score function:

$$\frac{\partial \log p(a|\Delta w)}{\partial \Delta w_{ij}} = -\frac{1}{\sigma^2} (\Delta w_{ij} - \mu_{ij}), \tag{19}$$

which characterizes the sensitivity of the log-likelihood to changes in the weight update.

B. Scaling property. We now derive the scaling behavior of the score function under multiplicative scaling of the weight update $\Delta w \to c\Delta w$, for a scalar $c > 0$. Here, we define a new variable $v = c \cdot \Delta w$, and examine $\log p(a|v)$. Substituting $\Delta w = \frac{1}{c}v$ into the log-likelihood:

$$\log p(a|v) = -\frac{1}{2\sigma^2}\left\|\frac{1}{c}v_{ij} - \mu_{ij}\right\|^2 + C'. \quad [20]$$

Differentiating with respect to $v_{ij} = c\Delta w_{ij}$, we apply the chain rule:

$$\frac{\partial \log p(a|v)}{\partial v_{ij}} = \frac{d}{dv_{ij}}\left(-\frac{1}{2\sigma^2}\left\|\frac{1}{c}v_{ij} - \mu_{ij}\right\|^2\right) \quad [21]$$

$$= -\frac{1}{\sigma^2} \cdot \frac{1}{c}\left(\frac{1}{c}v_{ij} - \mu_{ij}\right) \quad [22]$$

$$= \frac{1}{c} \cdot \left(-\frac{1}{\sigma^2}\left(\Delta w_{ij} - \mu_{ij}\right)\right) \quad [23]$$

$$= \frac{1}{c} \cdot \frac{\partial \log p(a|\Delta w)}{\partial \Delta w_{ij}}. \quad [24]$$

Thus, the score function scales inversely:

$$\frac{\partial \log p(a|c\Delta w)}{\partial (c\Delta w_{ij})} = \frac{1}{c} \cdot \frac{\partial \log p(a|\Delta w)}{\partial \Delta w_{ij}}. \quad [25]$$

with scalar $c > 0$. Setting $c = |\Delta w|$, we decompose Δw as $\Delta w = |\Delta w| \cdot \hat{w}$, where $\hat{w}$ is the unit vector in the direction of Δw. Thus, we obtain the following relation for the score function (Eq. 17):

$$\frac{\partial \log p(a \mid \Delta w)}{\partial \Delta w} = \frac{\partial \log p(a \mid |\Delta w| \cdot \hat{w})}{\partial (|\Delta w| \cdot \hat{w})} = \frac{1}{|\Delta w|} \cdot \frac{\partial \log p(a \mid \hat{w})}{\partial \hat{w}} \quad [26]$$

Since FIM is defined as the expected outer product of the score function (Eq. 16), and each component of the score scales as $\frac{1}{|\Delta w|}$, their outer product scales as $\frac{1}{|\Delta w|^2}$. Thus, we derive the scaling form:

$$F_{kl}(\Delta w) = \frac{1}{|\Delta w|^2}G_{kl}, \quad [27]$$

where G_{kl} is a direction-dependent matrix independent of the magnitude $|\Delta w|$. This scaling property of FIM (Eq. 27) governs the form of the Kullback-Leibler divergence, which will be demonstrated in the following derivation.

III. Derivation of Kullback-Leibler divergence

A. Second-order approximation. The Kullback-Leibler (KL) divergence between two probability distributions $p(a|\Delta w)$ and $p(a)$ measures the information gain about activity provided by observing weight update:

$$D_{KL}(p(a|\Delta w)||p(a)) = \int p(a|\Delta w)\log\frac{p(a|\Delta w)}{p(a)}da. \quad [28]$$

For small perturbation δ in the parameter space, the KL divergence can be approximated using FIM (7):

$$D_{KL}(p(a|\Delta w)||p(a|\Delta w + \delta)) = \frac{1}{2}\delta^T F(\Delta w)\delta + o(||\delta||^2), \quad [29]$$

which is a second-order approximation for small δ. To compute the KL divergence between $p(a|\Delta w)$ and $p(a)$, we consider the special case where $p(a) = p(a|\Delta w = 0)$, i.e., the distribution of activity in the absence of weight update. With this assumption, we can view $D_{KL}(p(a|\Delta w)||p(a))$ as a special case of $D_{KL}(p(a|\Delta w)||p(a|\Delta w+\delta))$ with $\delta = -\Delta w$. Note that the approximation in Eq. 29 implicitly assumes that the weight update direction is aligned with the gradient of the log-likelihood, which naturally holds in Stochastic Gradient Descent (SGD). However, adaptive methods like Adam rescale each coordinate of the gradient differently, which may result in altering the update direction and breaking its alignment with the original gradient.

B. Path Integral Formulation. We can compute $D_{KL}(p(a|\Delta w)||p(a))$ by integrating the infinitesimal contributions along a path from $\Delta w = 0$ to Δw:

$$D_{KL}(p(a|\Delta w)||p(a)) = \int_\gamma \nabla_{\Delta w'} D_{KL}(p(a|\Delta w')||p(a)) \cdot d\Delta w', \quad [30]$$

where γ is a path from 0 to Δw. Here we parameterize it as:

$$\gamma(t) = t \cdot \Delta w, \quad 0 \le t \le 1, \quad [31]$$

then, by change of variables, we rewrite the integral as:

$$D_{KL}(p(a|\Delta w)||p(a)) = \int_0^1 \nabla_{\gamma(t)} D_{KL}(p(a|\gamma(t))||p(a)) \cdot \frac{d\gamma(t)}{dt} dt. \quad [32]$$

Considering $\frac{d\gamma(t)}{dt} = \Delta w$, this becomes:

$$D_{KL}(p(a|\Delta w)||p(a)) = \int_0^1 \nabla_{\gamma(t)} D_{KL}(p(a|t\Delta w)||p(a)) \cdot \Delta w \, dt. \quad [33]$$

To compute the integrand, we note that the KL divergence can be locally approximated as a quadratic form near $\Delta w = 0$ using FIM (Eq. 29), which leads to the gradient:

$$\nabla_\gamma D_{KL}(p(a|\gamma)||p(a)) \approx F(\gamma) \cdot \gamma. \quad [34]$$

This local quadratic approximation has been validated by our numerical simulations (Fig. S8). Applying the chain rule with $\gamma(t) = t\Delta w$, we obtain:

$$\frac{d}{dt} D_{KL}(p(a|t\Delta w)||p(a)) = \nabla_{\gamma(t)} D_{KL}(p(a|\gamma(t))||p(a)) \cdot \frac{d\gamma(t)}{dt} \quad [35]$$

$$= (F(t\Delta w) \cdot t\Delta w)^T \cdot \Delta w \quad [36]$$

$$= (t\Delta w)^T F(t\Delta w)\Delta w, \quad [37]$$

where the transpose ensures a scalar result by forming a valid inner product between column vectors. Substituting the FIM equation $F(t\Delta w)$ using scaling form in Eq. 27:

$$\frac{d}{dt} D_{KL}(p(a|t\Delta w)||p(a)) = (t\Delta w)^T \cdot \frac{1}{t^2|\Delta w|^2} G \cdot \Delta w \quad [38]$$

$$= \frac{t\Delta w^T \cdot G \cdot \Delta w}{t^2|\Delta w|^2} \quad [39]$$

$$= \frac{1}{t} \cdot \frac{\Delta w^T G \Delta w}{|\Delta w|^2}. \quad [40]$$

We denote the direction-dependent term as $g(\theta) = \frac{\Delta w^T G \Delta w}{|\Delta w|^2}$, then

$$\frac{d}{dt} D_{KL}(p(a|t\Delta w)||p(a)) = \frac{g(\theta)}{t}. \quad [41]$$

To properly handle the singularity at $t = 0$, we introduce a small cutoff $\epsilon > 0$ and compute

$$D_{KL}(p(a|\Delta w)||p(a)) = \int_\epsilon^1 \frac{d}{dt} D_{KL}(p(a|t\Delta w)||p(a)) dt = \int_\epsilon^1 \frac{g(\theta)}{t} dt. \quad [42]$$

To regularize the divergence at $t = 0$, we introduce a lower bound ϵ on the path parameter t. Without assuming any specific form of the KL divergence, we require that the smallest resolvable perturbation along the path, given by $\epsilon \cdot |\Delta w|$, corresponds to a fixed reference scale $|\Delta w_0|$ below which the Fisher approximation may become invalid. Accordingly, we define $\epsilon = \frac{|\Delta w_0|}{|\Delta w|}$ and evaluate the integral as

$$D_{KL}(p(a|\Delta w)||p(a)) = g(\theta) \int_\epsilon^1 \frac{1}{t} dt = g(\theta) \log\left(\frac{1}{\epsilon}\right) \quad [43]$$

$$= g(\theta) \log\left(\frac{|\Delta w|}{|\Delta w_0|}\right) = \beta \log|\Delta w| + C_w, \quad [44]$$

where $\beta = g(\theta)$ is a direction-dependent constant and $C_w = -\beta \log|\Delta w_0|$ is a direction-dependent constant offset determined by the choice of reference scale $|\Delta w_0|$.

IV. Maximum entropy principle with mutual information constraint

The maximum entropy principle (8) states that the most likely distribution is the one that maximizes entropy while satisfying given constraints. We consider maximizing entropy while satisfying the mutual information constraint between weight update and activity:

$$\max H(p(\Delta w)) \text{ s.t. } I(\Delta w; a) = C_I, \quad [45]$$

where C_I is a constant for mutual information constraint, and the entropy H is defined as:

$$H(p(\Delta w)) = -\int p(\Delta w) \log p(\Delta w) d(\Delta w). \quad [46]$$

The mutual information I between weight update Δw and activity a is:

$$I(\Delta w; a) = \int\int p(\Delta w, a) \log \frac{p(\Delta w, a)}{p(\Delta w)p(a)} d(\Delta w) da. \quad [47]$$

To establish the connection with the KL divergence, we apply a Bayesian transformation using the conditional probability formula: $p(\Delta w, a) = p(\Delta w)p(a|\Delta w)$, then the mutual information I can be expressed as:

$$\begin{aligned} I(\Delta w; a) &= \int\int p(\Delta w)p(a|\Delta w) \log \frac{p(\Delta w)p(a|\Delta w)}{p(\Delta w)p(a)} d(\Delta w) da \\ &= \int\int p(\Delta w)p(a|\Delta w) \log \frac{p(a|\Delta w)}{p(a)} d(\Delta w) da \\ &= \int p(\Delta w) \left[\int p(a|\Delta w) \log \frac{p(a|\Delta w)}{p(a)} da\right] d(\Delta w) \\ &= \int p(\Delta w) D_{KL}(p(a|\Delta w)||p(a)) d(\Delta w). \end{aligned} \quad [48]$$

Using the method of Lagrange multipliers:

$$\mathcal{L} = -\int p(\Delta w) \log p(\Delta w) d(\Delta w) \quad [49]$$

$$- \lambda \left(\int p(\Delta w) D_{KL}(p(a|\Delta w)||p(a)) d(\Delta w) - C_I\right) \quad [50]$$

$$- \mu \left(\int p(\Delta w) d(\Delta w) - 1\right), \quad [51]$$

where λ is the Lagrange multiplier associated with the mutual information constraint, and μ corresponds to the normalization condition. Then we take the functional derivative with respect to $p(\Delta w)$ and set it to zero:

$$\frac{\delta \mathcal{L}}{\delta p(\Delta w)} = -\log p(\Delta w) - 1 - \lambda D_{KL}(p(a|\Delta w)||p(a)) - \mu = 0. \quad [52]$$

Solving for $p(\Delta w)$ yields:

$$p(\Delta w) = e^{(-1-\mu-\lambda D_{KL}(p(a|\Delta w)||p(a)))}. \quad [53]$$

By substituting the logarithmic form of the KL divergence given in Eq. 44, we obtain:

$$p(\Delta w) = e^{(-1-\mu-\lambda\beta \log|\Delta w| - \lambda C_w)} \quad [54]$$

$$= e^{(-1-\mu-\lambda C_w)} \cdot |\Delta w|^{-\lambda\beta}. \quad [55]$$

Let $Z = e^{(1+\mu+\lambda C_w)}$ be the normalization constant and $\alpha = \lambda\beta$, we arrive at:

$$p(\Delta w) = \frac{1}{Z}|\Delta w|^{-\alpha}, \quad [56]$$

which corresponds to a scaling law distribution. Here, λ is the Lagrange multiplier associated with the mutual information constraint, and β is a direction-dependent constant.

Over the course of training (e.g., during a training period denoted by $\tilde{t}$), the mutual information $I_{\tilde{t}}(\Delta w; a)$ between weight updates and target activity tends to increase or remain stable (Fig. 3 and Figs. S9 and S12). Accordingly, the Lagrange multiplier λ also increases or remains unchanged to ensure the mutual information constraint is satisfied. Specifically, we assume that λ is a monotonic function of the mutual information, i.e.,

$$\lambda = g(I(\Delta w; a)) \quad [57]$$

where $g(\cdot)$ is a monotonic function. Hence, we identify a generalized relationship that holds across different network architectures and training tasks:

$$\alpha_{\tilde{t}} \propto g(I_{\tilde{t}}(\Delta w; a)) \cdot \beta_{\tilde{t}}, \quad [58]$$

where $\beta_{\tilde{t}}$ captures direction-dependent variability in the gradients, independent of weight magnitudes.

V. Optimizer families and update formulas

To highlight the relation between standard gradient-based and adaptive gradient-based methods, we summarize the update rules of the two major optimizer families: SGD-family and Adaptive-family. The SGD-family, which includes SGD and Nesterov Accelerated Gradient (9) (NAG), shares a common form of direct gradient-based update with optional momentum. In contrast, the Adaptive-family, which includes Adaptive Moment Estimation (10) (Adam), Root Mean Square Propagation (11) (RMSProp), and the Adaptive Gradient Algorithm (12) (AdaGrad), incorporates parameter-wise normalization based on accumulated gradient statistics to adjust the step size for each parameter. The update formulas are provided in Tables S1-S2, and the probability and complementary cumulative distributions of updates are shown in Figs. S6-S7.

Table S1. Comparison of SGD-family and Adaptive-family optimizers.

Optimizer	Update formula	Characteristics
**SGD-family**		
**SGD**	$w_{t+1} = w_t - r\nabla L_t$	Gradient descent with fixed step size (learning rate r).
**NAG**	$v_{t+1} = \mu v_t - r\nabla L(w_t + \mu v_t),$<br>$w_{t+1} = w_t + v_{t+1}$	NAG introduces a momentum-based lookahead, which induces temporal correlations between consecutive updates and tends to yield less steep update tails compared to SGD.
**Adaptive-family**		
**Adam**	$m_t = \beta_1 m_{t-1} + (1-\beta_1)g_t,$<br>$v_t = \beta_2 v_{t-1} + (1-\beta_2)g_t^2,$<br>$w_{t+1} = w_t - r\dfrac{m_t/(1-\beta_1^t)}{\sqrt{v_t/(1-\beta_2^t)}+\epsilon'}$	Adam combines momentum and adaptive normalization, yielding heavy-tailed updates whose large exploratory updates are truncated by per-parameter normalization.
**RMSProp**	$E[g^2]_t = \rho E[g^2]_{t-1} + (1-\rho)g_t^2,$<br>$w_{t+1} = w_t - \dfrac{r}{\sqrt{E[g^2]_t + \epsilon'}} g_t$	RMSProp uses an exponential moving average of squared gradients, thereby adaptively rescaling the effective learning step while retaining moderately heavy-tailed update statistics.
**AdaGrad**	$G_t = \sum_{\tau=1}^{t} g_\tau^2,$<br>$w_{t+1} = w_t - \dfrac{r}{\sqrt{G_t + \epsilon'}} g_t$	AdaGrad accumulates squared gradients over time to normalize each update, reducing the step size as training proceeds, thereby suppressing large updates and producing rapidly decaying tails.

Note: The symbol v_t denotes different quantities across optimizers: in NAG, it represents the velocity (momentum term), whereas in Adam it denotes the exponential moving average of squared gradients.

Table S2. Definitions of symbols used in optimizer update formulas.

Symbol	Definition
w_t	Model parameters at iteration t
$g_t = \nabla L_t$	Gradient of the loss function with respect to w_t
r	Global learning rate or step size
μ	Momentum coefficient in Nesterov Accelerated Gradient (NAG)
ρ	Decay rate for the exponential moving average of squared gradients (RMSProp)
β_1, β_2	Exponential decay rates for first- and second-moment estimates (Adam)
m_t	First-moment (momentum) estimate in Adam
v_t	Second-moment (variance) estimate in Adam
G_t	Accumulated sum of squared gradients in AdaGrad
ϵ'	Small constant for numerical stability

VI. Supplementary Figures

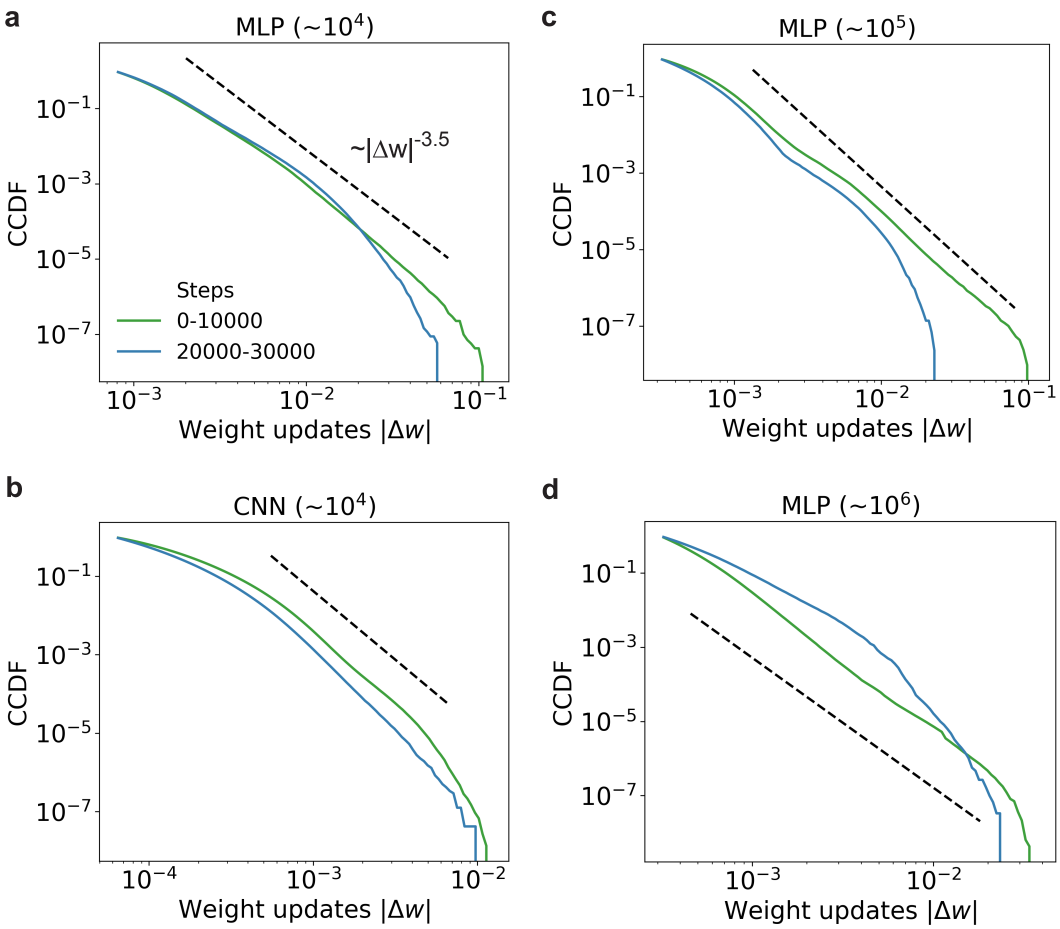

Fig. S1. Complementary cumulative distribution functions (CCDFs) of update magnitudes across models, scales, and training stages. (a-d), The CCDFs of weight updates ($|\Delta w|$) are shown on log–log scales corresponding to the probability distributions in Fig. 2 (main text). The CCDFs were computed from the first logarithmic bin (excluding near-zero updates) to reduce numerical noise and enhance the visibility of the tail region. Green and blue curves denote rapid-loss (steps 0 to 10,000) and near-convergence (steps 20,000 to 30,000) training stages, respectively. Across all configurations, the CCDFs exhibit nearly linear behavior in the intermediate range, confirming heavy-tailed statistics with indicative slopes of $|\Delta w|^{-(\alpha-1)}$ (where $\alpha = 4.5$ in Fig. 2). The exponent decreases by one relative to the probability distribution because the CCDF represents the integrated tail probability, $P(|\Delta w| > x) \propto \int_x^\infty |\Delta w|^{-\alpha}\, d|\Delta w| \sim |\Delta w|^{-(\alpha-1)}$.

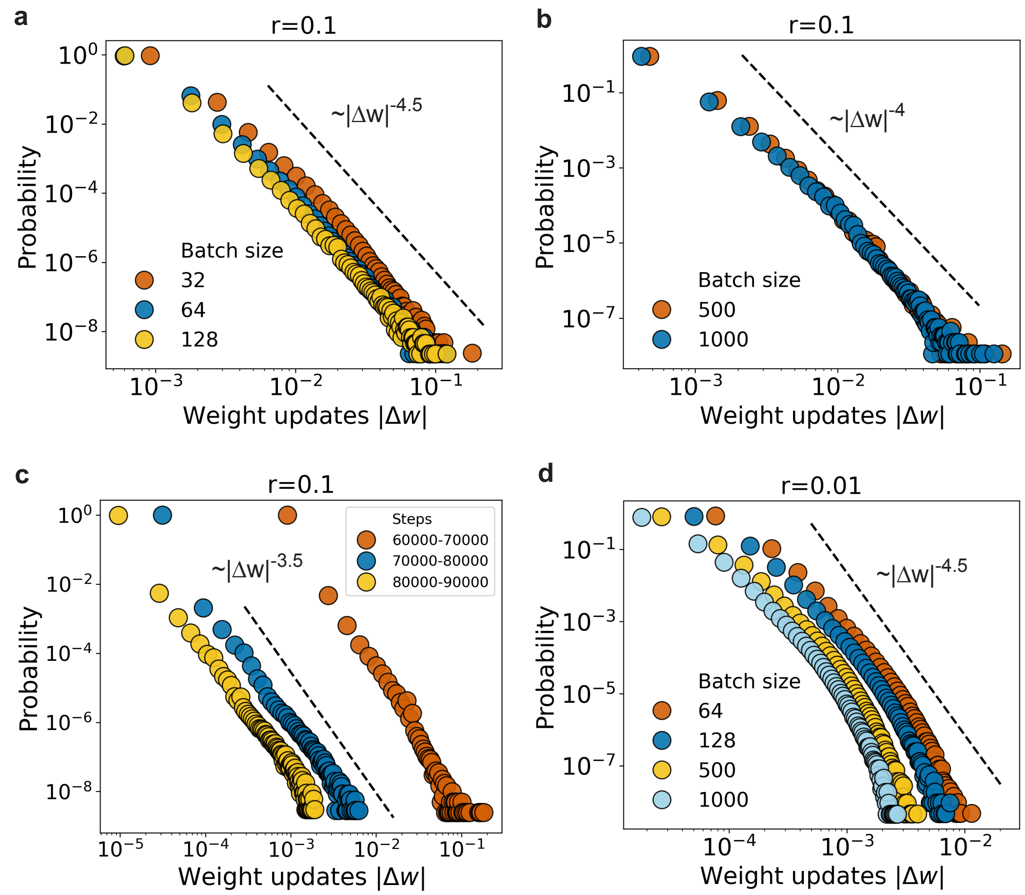

Fig. S2. Robustness of heavy-tailed update distributions across batch sizes and training stages. a, b, Update distributions for MLPs ($\sim 10^4$) trained on the MNIST dataset with a learning rate ($r = 0.1$) under varying batch sizes. Small batch sizes (32, 64, 128) correspond to panel **a** (steps 0 to 10,000), while larger batch sizes (500, 1000) correspond to panel **b** (steps 0 to 5,000). In both cases, the updates $|\Delta w|$ exhibit scaling laws with varying exponents (e.g., from 3 to 5), indicating that heavy-tailed behavior persists regardless of mini-batch sizes. **c**, Evolution of update distributions over different training stages (steps 60,000 to 90,000) under $r = 0.1$, showing that the heavy-tailed nature is preserved over longer training stages. **d**, Update distributions under a smaller learning rate ($r = 0.01$) across a broad range of batch sizes (64 to 1000). The distribution remains heavy-tailed with an exponent around 4.5, further confirming the universality of such scaling behavior across hyperparameter configurations.

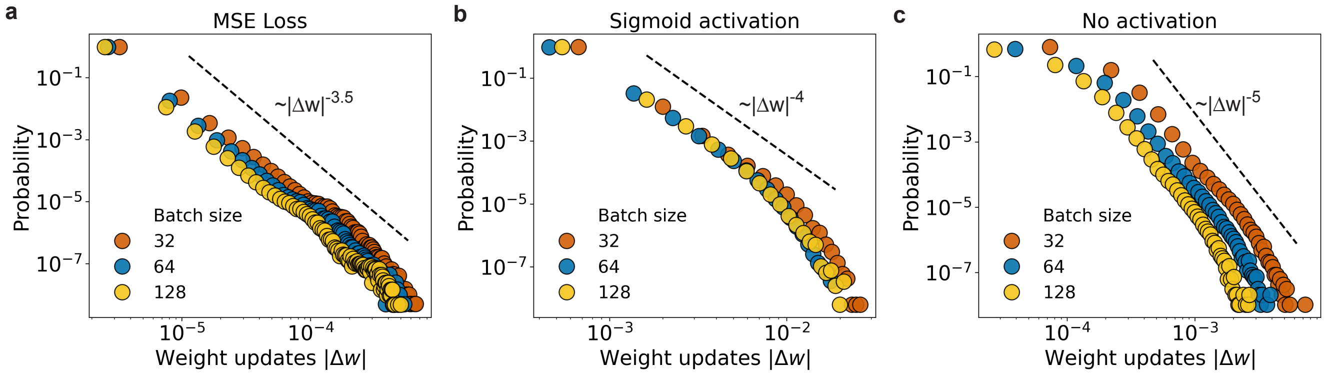

Fig. S3. Heavy-tailed update distributions under alternative loss and activation functions. **a**, Update distributions for MLPs ($\sim 10^4$) trained with mean squared error (MSE) loss and varying batch sizes (e.g., 32, 64, 128), using a learning rate of $r = 0.01$, exhibit heavy-tailed scaling with an exponent of approximately 3.5, indicating that the heavy-tailed property is preserved even under a regression-style loss function. **b**, Using a Sigmoid activation function instead of ReLU also results in a power-law-like distribution, with a steeper exponent of approximately 4 across batch sizes, using a learning rate of $r = 0.1$. **c**, Results without an activation function exhibit a power-law-like distribution with an indicative exponent of approximately 5, also under $r = 0.1$. These results demonstrate that the emergence of heavy-tailed update statistics is robust to variations in both the loss function and the activation function.

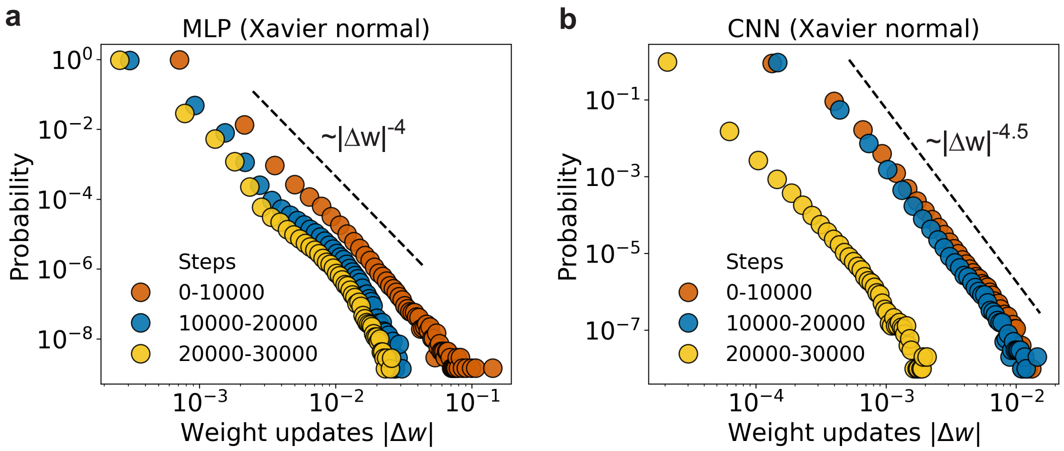

Fig. S4. Effect of Xavier normal initialization on update distributions. Log-log plots of update magnitudes $|\Delta w|$ distributions during training under Xavier normal initialization (13). **a**, MLP ($\sim 10^5$) with learning rate $r = 0.1$; **b**, CNN ($\sim 10^4$) with learning rate $r = 0.01$. Updates are collected over three training intervals: early (steps 0 to 10,000), mid (steps 10,000 to 20,000), and late phases (steps 20,000 to 30,000). Both models were initialized using Xavier (Glorot) normal initialization, in contrast to our default initialization scheme, Kaiming uniform initialization (14). Despite the change in initialization, the update distributions remain heavy-tailed throughout training with power-law exponents near 4 to 4.5.

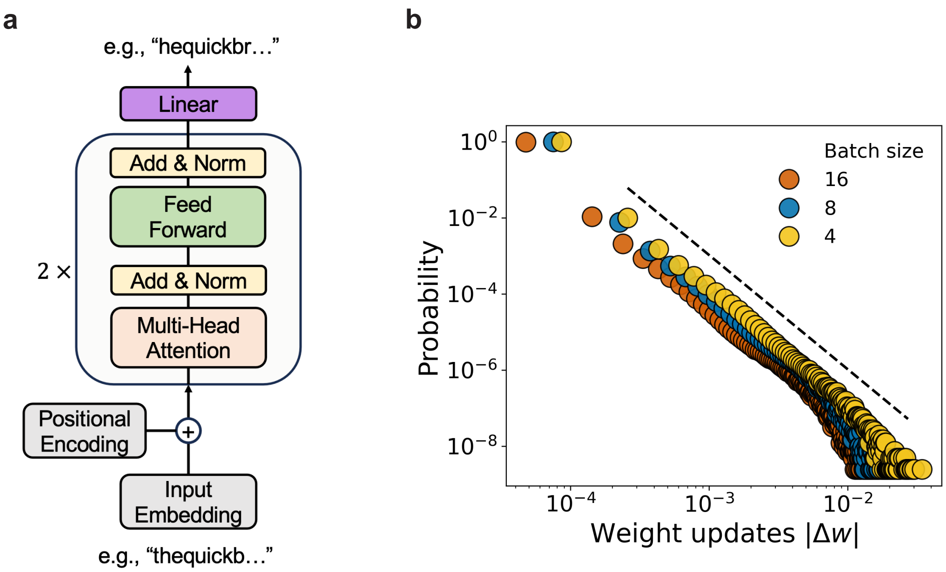

Fig. S5. **Character-level Transformer model and heavy-tailed updates. a**, Schematic of the simplified Transformer encoder used in our analysis. Complementary to results obtained on MLP and CNN architectures, we adopt a character-level Transformer consisting of an input embedding layer, positional encoding, and a two-layer Transformer encoder block where each including 4-head self-attention and feed-forward layers, with residual connections and layer normalization. The model is trained on a repeated 26-character corpus at the character level (`"thequickbrownfoxjumpsoverthelazydog"` $\times$ 100). It takes as input a continuous character stream (e.g., `"thequickb..."`) and learns to predict the next character in the sequence (e.g., `"hequickbr..."`), effectively modeling sequential dependencies in text. **b**, Empirical distribution of weight update magnitudes $|\Delta w|$ during training using stochastic gradient descent (SGD) with learning rate $r = 0.1$. The update distribution follows a heavy-tailed power-law with an exponent of approximately $|\Delta w|^{-3}$ (the dashed indicative line), consistent across different batch sizes (4, 8, 16). This observation highlights that even in the Transformer-based architecture, criticality-like behavior in parameter update dynamics emerges under training.

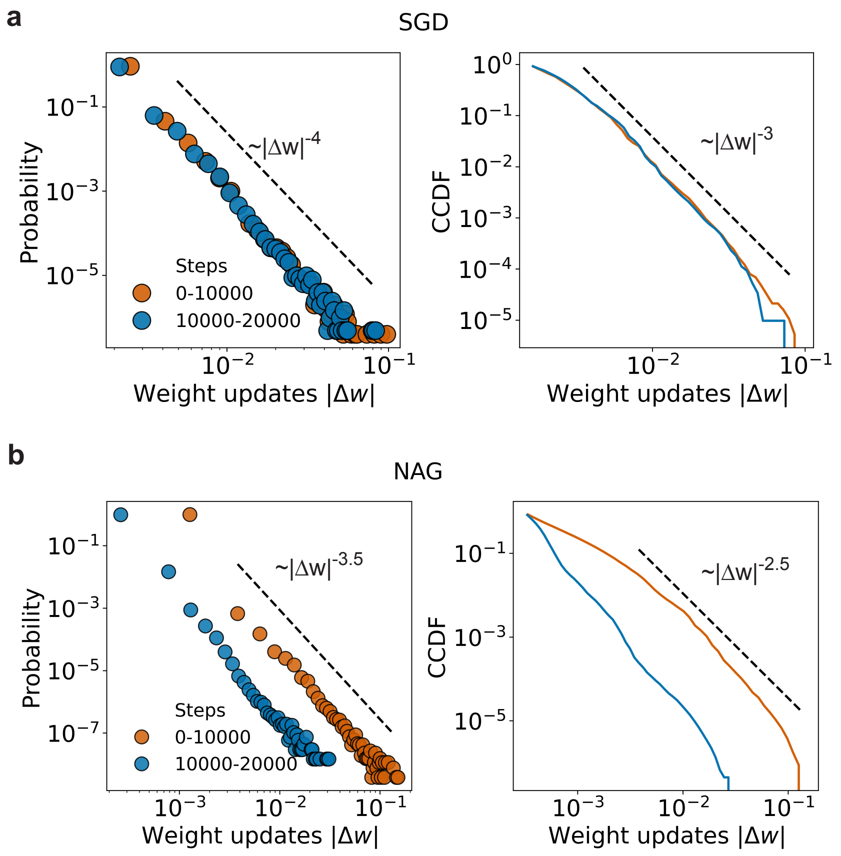

Fig. S6. Heavy-tailed update distributions of standard gradient-based optimizers under full-batch training. (a, b) Update distributions (left) and corresponding complementary cumulative distribution functions (CCDFs; right) for SGD and Nesterov Accelerated Gradient (NAG), measured over two consecutive training step windows (steps 0 to 10,000 and steps 10,000 to 20,000). Updates were collected during the training of a five-layer MLP ($\sim 10^4$ parameters) under full-batch learning with a learning rate of $r = 0.01$ on the MNIST dataset. The CCDFs were computed beginning from the first logarithmic bin, excluding near-zero updates, to reduce numerical noise and highlight the tail behavior. Both optimizers exhibit heavy-tailed update distributions across training stages, with indicative power-law slopes of approximately -4 and -3.5. Compared to SGD, NAG shows a slightly shallower tail, indicating a higher probability of large updates. This behavior arises because the momentum term in NAG introduces temporal correlations between successive updates, amplifying gradients and sustaining larger update magnitudes. If the updates follow a power-law $p(|\Delta w|) \sim |\Delta w|^{-\alpha}$, the corresponding CCDF scales as $|\Delta w|^{-(\alpha-1)}$, producing a slope one unit smaller than that of the probability distribution.

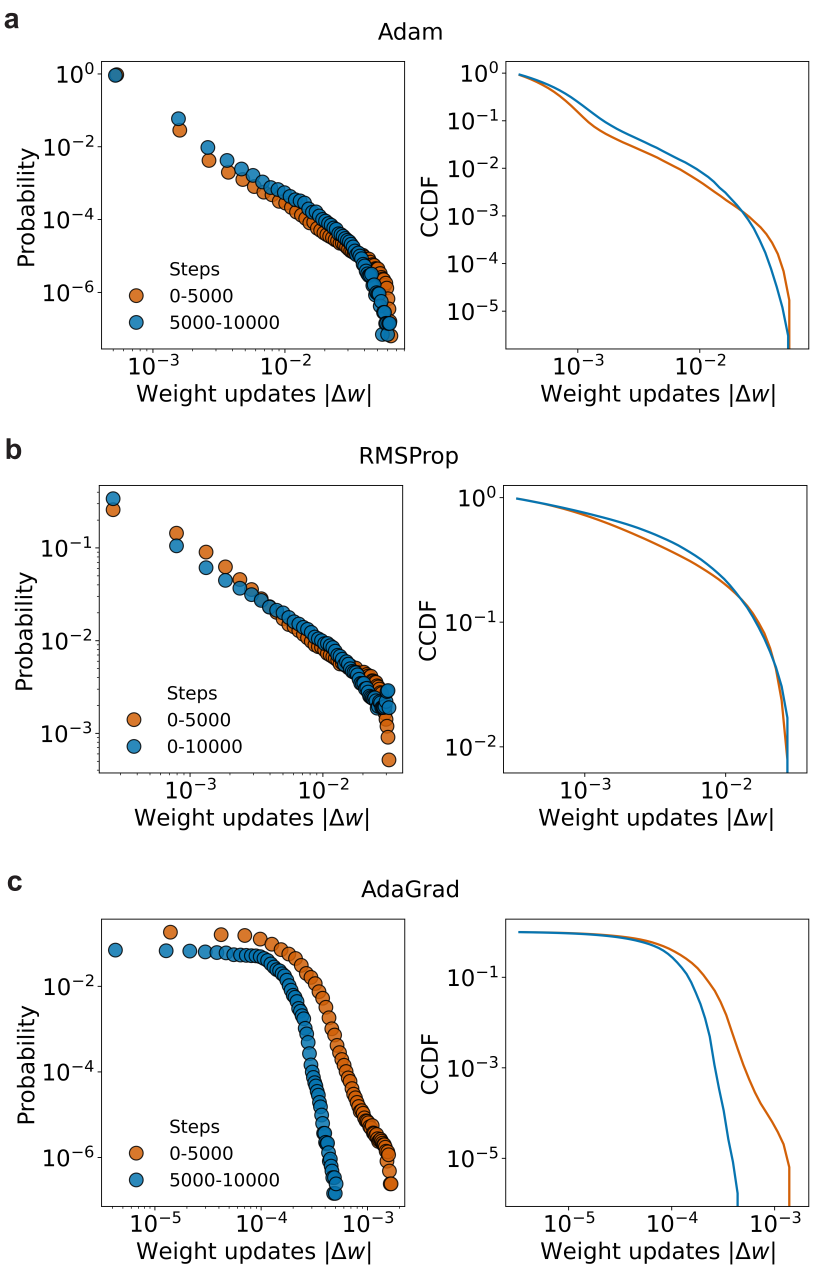

Fig. S7. **Update distributions of adaptive gradient-based optimizers under full-batch training. (a-c)** Update distributions (left) and complementary cumulative distribution functions (CCDFs; right) for Adaptive Moment Estimation (Adam; **a**), Root Mean Square Propagation (RMSProp; **b**), and Adaptive Gradient Algorithm (AdaGrad; **c**), measured over rapid-loss (steps 0 to 5,000) and near-convergence (steps 5,000 to 10,000) training stages. The CCDFs were computed from the first non-empty logarithmic bin, excluding near-zero updates to minimize numerical noise. Updates were collected from a five-layer MLP ($\sim 10^4$ parameters, full-batch, $r = 0.01$) trained on MNIST. Adaptive methods, such as Adam and RMSProp, maintain heavy-tailed update distributions, but their tails exhibit algorithmic cutoffs compared to SGD-family methods, reflecting adaptive normalization-induced suppression of large exploratory updates. In contrast, AdaGrad shows a rapidly decaying tail and a flatter small-update regime due to its cumulative variance normalization.

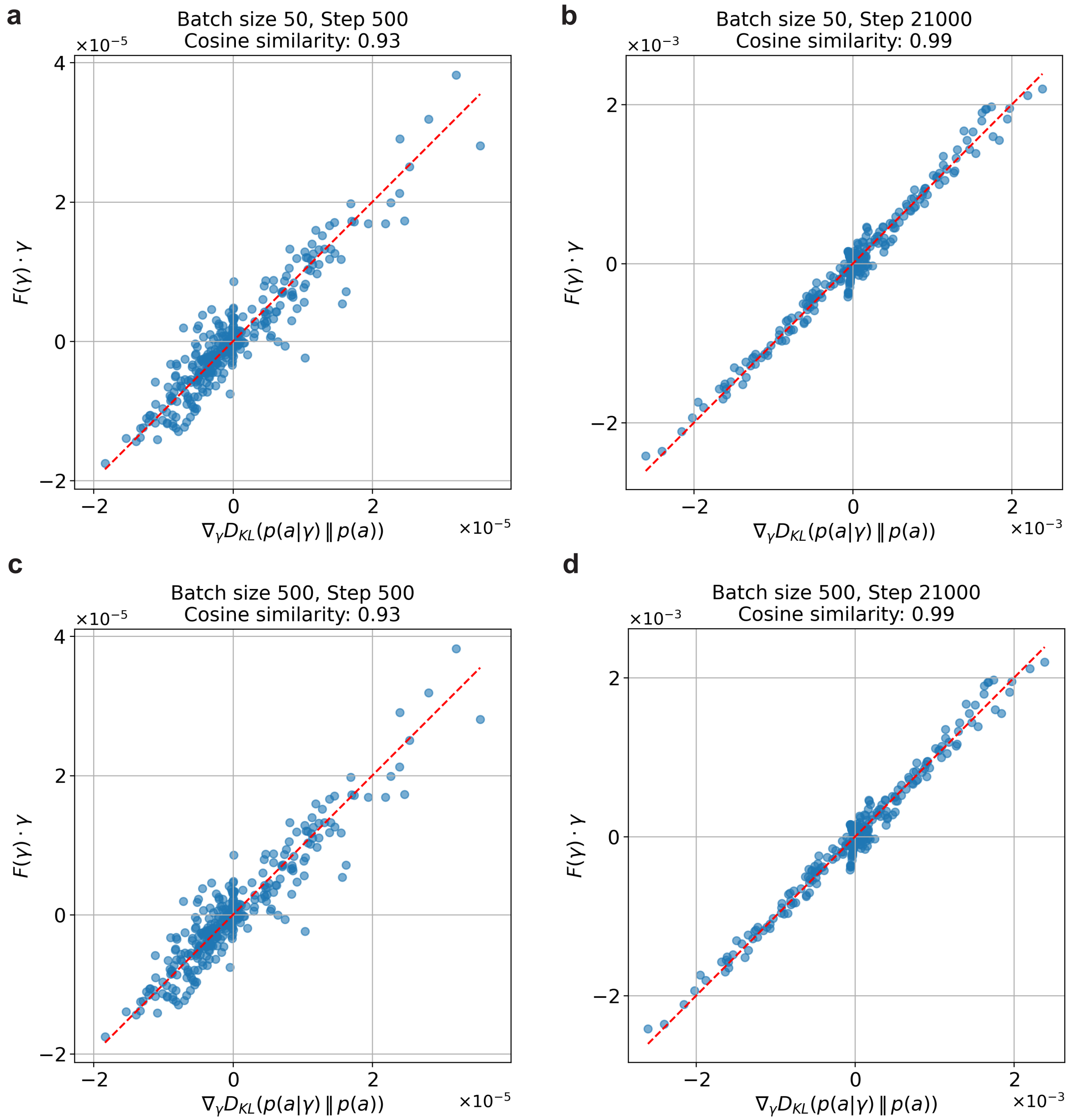

Fig. S8. Agreement between the true gradient of KL divergence and its Fisher approximation across training steps and batch sizes. Each panel shows a scatter plot comparing the true gradient of the KL divergence, $\nabla_\gamma D_{\mathrm{KL}}(p(a|\gamma)\,\|\,p(a))$, with the Fisher approximation, $F(\gamma)\cdot\gamma$, where $\gamma = \Delta w$ is the update from stochastic gradient descent (SGD). The model is a five-layer MLP trained on MNIST with learning rate $r = 0.01$, and evaluations are performed on the last layer. **a**, Training step 500 with batch size 50. **b**, Training step 21,000 with batch size 50. **c**, Training step 500 with batch size 500. **d**, Training step 21,000 with batch size 500. The red dashed line represents the identity line, indicating a perfect agreement. Cosine similarity is shown in each panel to quantify the alignment between the two quantities. Results show that the Fisher approximation closely matches the true gradient, especially at later training stages and with larger batch sizes.

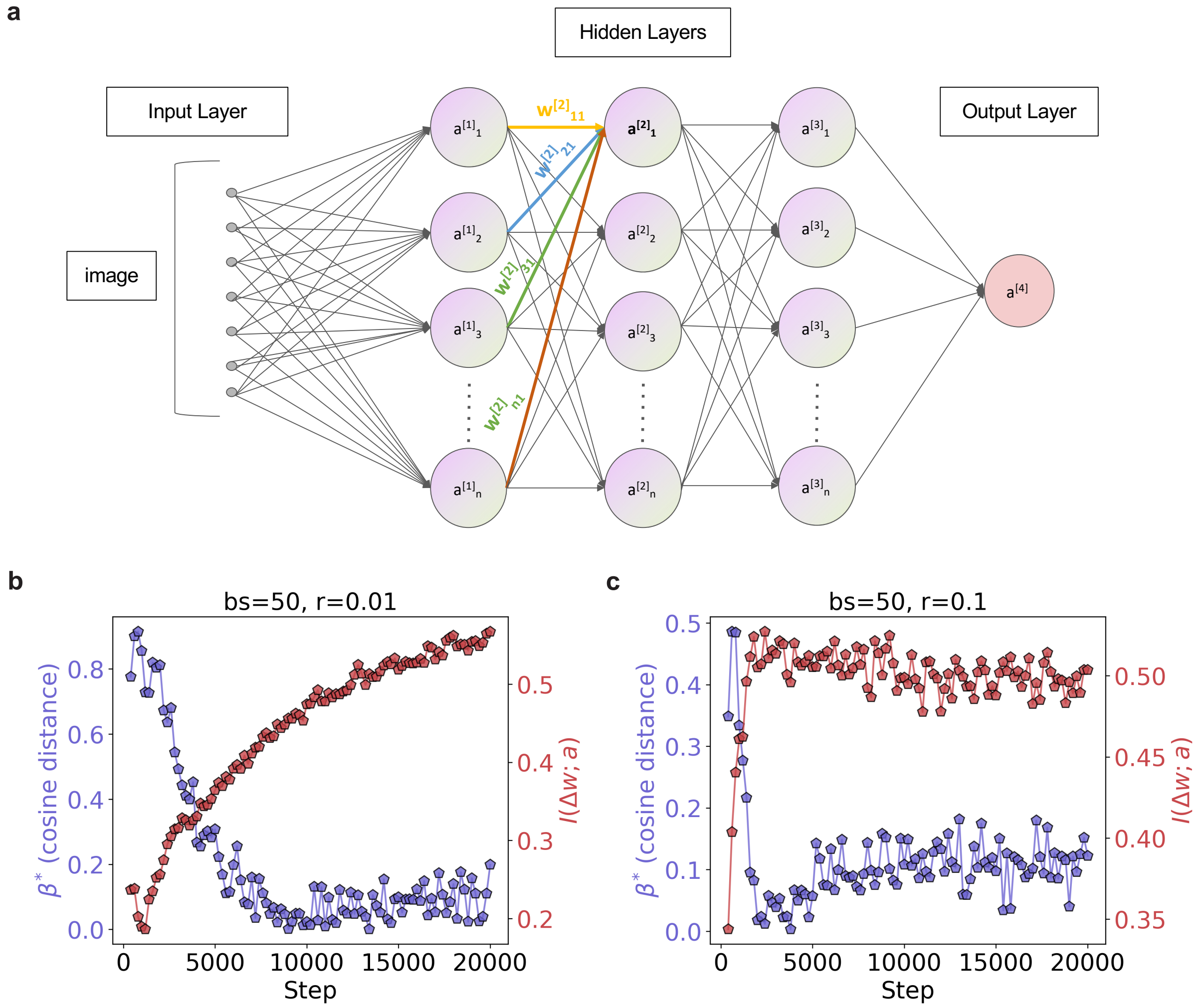

Fig. S9. **Estimated mutual information and gradient direction dynamics during training. a**, Illustration of the MLP architecture with four layers. Each edge in such neural network denotes a trainable weight $w_{ij}^{[\ell]}$, and $a_j^{[\ell]}$ denotes the activation of unit j at layer ℓ. Mutual information $I(\Delta w; a)$ is computed between the weight updates Δw and their corresponding unit activations a. Updates and activations from all fully connected layers are flattened and concatenated before analysis. **b, c**, Evolution of mutual information $I(\Delta w; a)$ (red, right axis) and cosine-based gradient direction β^* (blue, left axis) over training steps, under two learning rates: $r = 0.01$ (b) and $r = 0.1$ (c), with batch size of 50. Gradient direction is approximated by the cosine distance between the PCA-based principal components of updates in adjacent step intervals. This result shows that at a lower learning rate (b), both mutual information (MI) and direction (β) evolve gradually, whereas at a higher learning rate (c), MI and β stabilize rapidly.

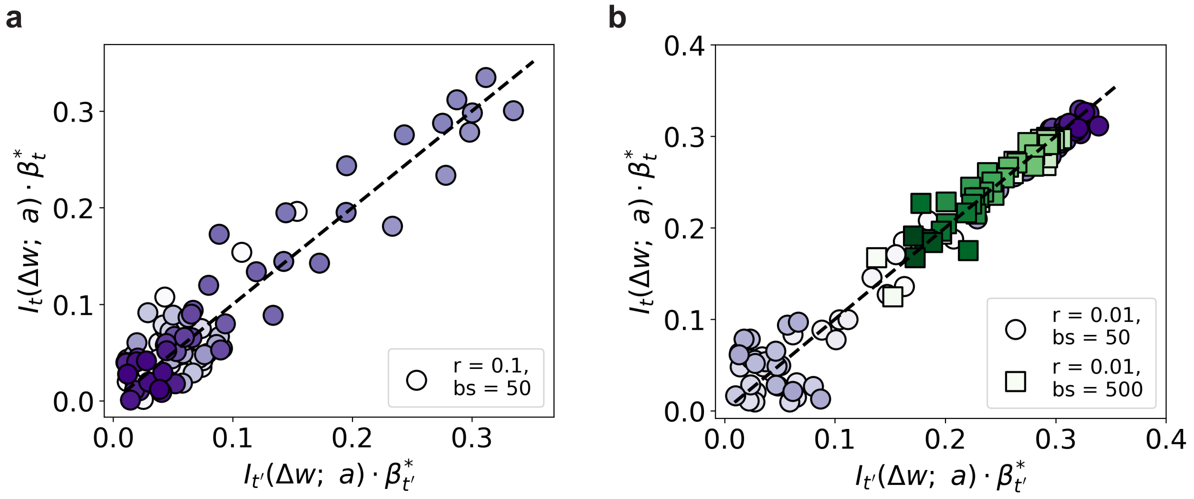

Fig. S10. Stability of the proxy for the power-law exponent across training steps and configurations. a, b, Empirical proxy of the power-law exponent $\alpha_t = g(I_t) \cdot \beta_t$ evaluated via the product $I_t(\Delta w; a) \cdot \beta^*_t$, compared against a later step, $I_{t'}(\Delta w; a) \cdot \beta^*_{t'}$. Each point represents a training step interval, with the marker color indicating training progress (darker colors correspond to later steps). A dashed identity line is shown to illustrate correspondence between successive proxy values. **a**, Configuration with learning rate $r = 0.1$ and batch size $bs = 50$ under $t' = t + 1$. The Spearman correlation coefficient between $I_t \cdot \beta^*_t$ and $I_{t'} \cdot \beta^*_{t'}$ is 0.75. **b**, Comparison of two batch sizes ($bs = 50$ and $bs = 500$) under fixed learning rate $r = 0.01$ under $t' = t + 1$. The strong temporal consistency of the proxy (Spearman correlation coefficients of 0.94 for both batch sizes $bs = 50$ and $bs = 500$) indicates that the product $I \cdot \beta^*$ remains approximately invariant across nearby training steps, supporting the stability of the heavy-tailedness of power-law-like distributions under different training regimes.

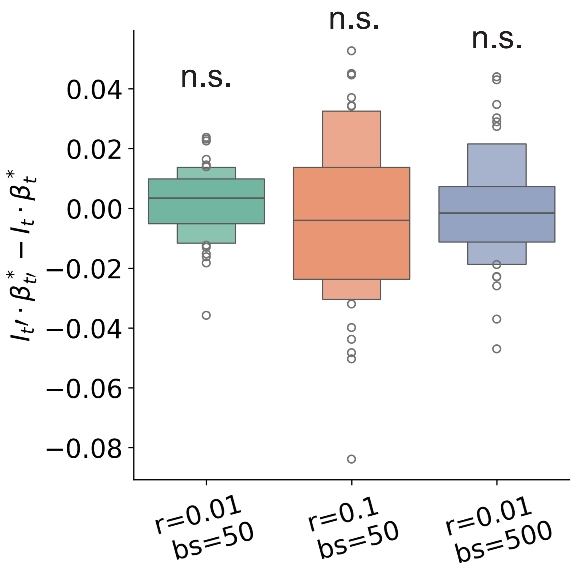

Fig. S11. **Stability of estimated exponents in the learning process.** Boxen plot showing the differences in estimated power-law exponents between adjacent steps, denoted as $I_{t'} \cdot \beta^*_{t'} - I_t \cdot \beta^*_t$, under different learning rates (r) and batch sizes (bs). Each box summarizes the distribution of temporal changes in $I(\Delta w; a) \cdot \beta^*$ throughout training, with the median, interquartile range, and whiskers indicating variability. Outliers are shown as individual points. Across all configurations, the differences remain small, indicating the evolution of heavy-tailedness over time with minor fluctuations. A two-sided one-sample t-test against zero found no statistically significant deviation across configurations $(p > 0.05)$, indicating that the estimated power-law exponent remains approximately invariant between adjacent steps, i.e., $I_{t'}(\Delta w; a) \cdot \beta^*_{t'} \approx I_t(\Delta w; a) \cdot \beta^*_t$.

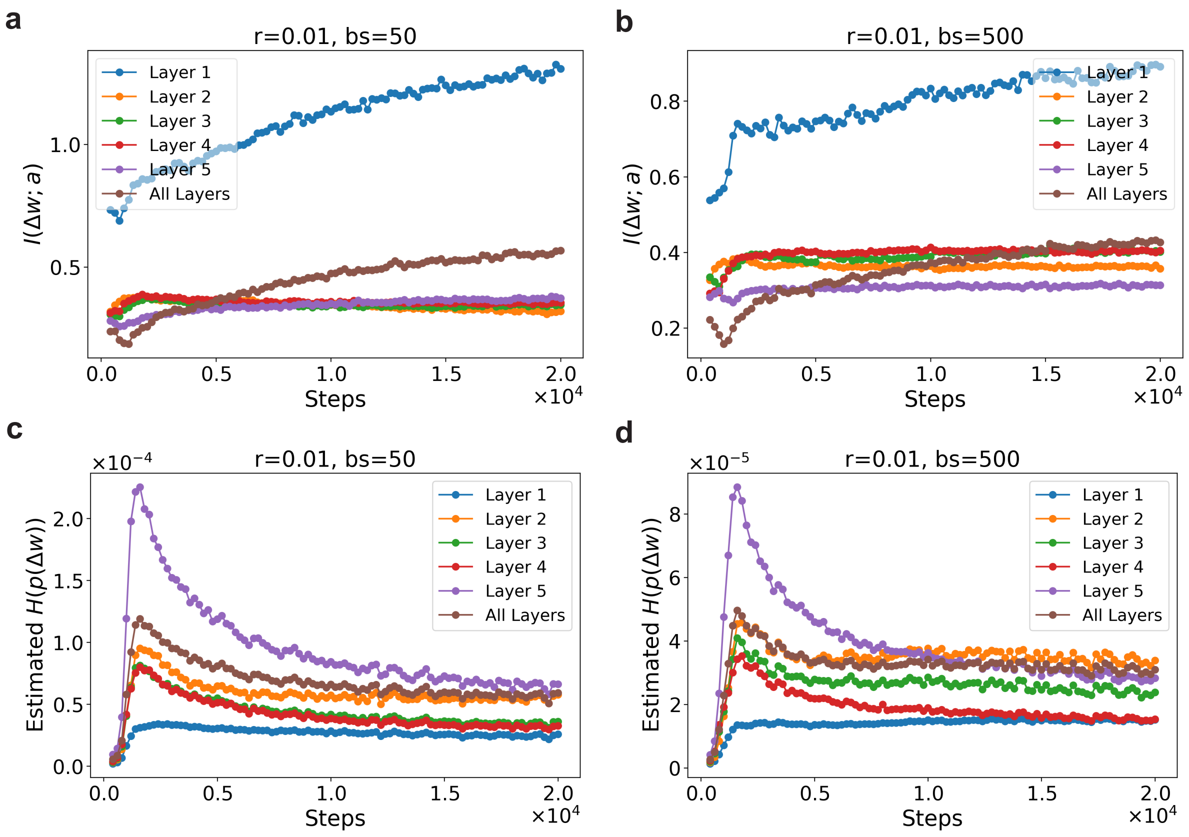

Fig. S12. Layer-wise mutual information and entropy evolution during training. a, b, Mutual information $I(\Delta w; a)$ between weight updates and activations, computed separately for each layer and the entire network, under batch sizes $bs = 50$ (a) and $bs = 500$ (b) with learning rate $r = 0.01$. A consistent upward trend is observed, particularly in the first layer, where the transfer of task-relevant information from inputs to activations is most prominent. **c, d**, Estimated entropy $H(p(\Delta w))$ of weight updates under the same training conditions. Entropy increases during the early stages of training, then decreases and eventually stabilizes, reflecting a non-monotonic transition from exploratory dynamics to more constrained update behavior. The observed competitive interplay between entropy and mutual information supports our theoretical framework: higher entropy implies weaker task-relevant information constraints, while lower entropy corresponds to more information-driven adaptation.

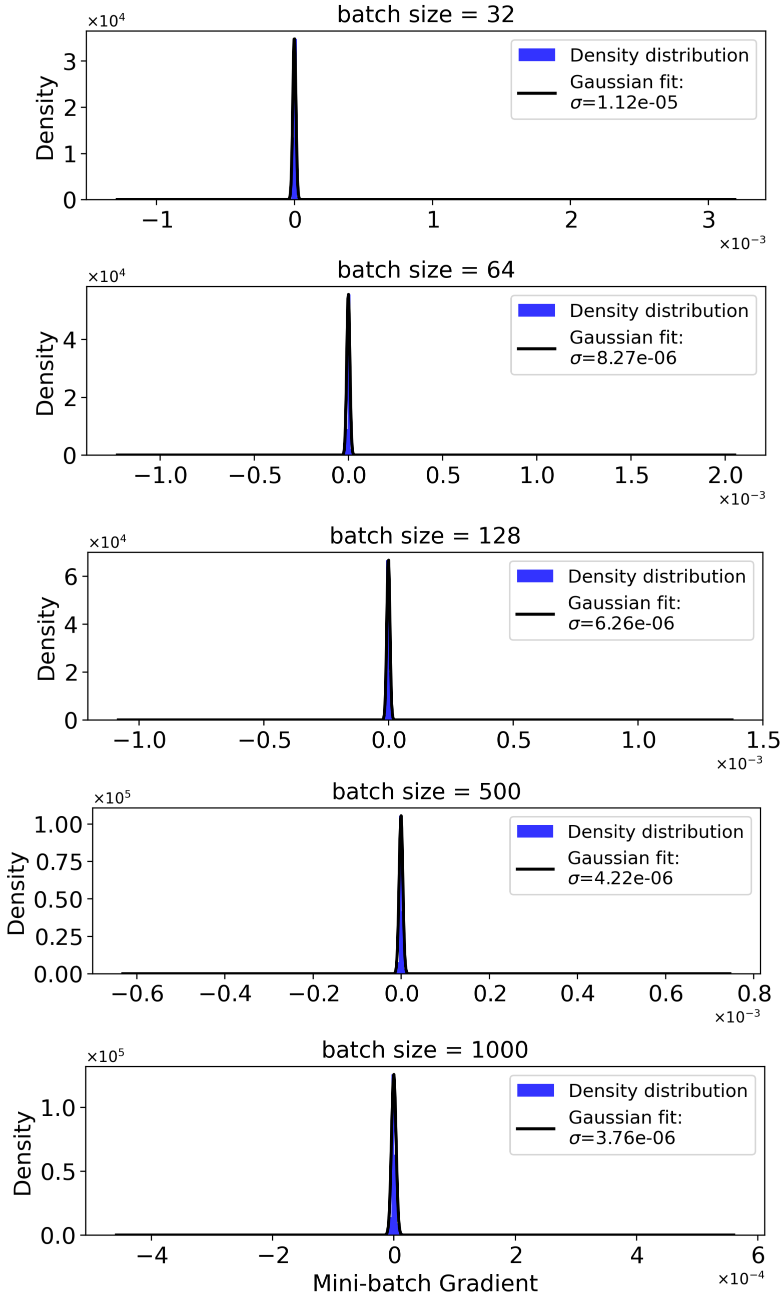

Fig. S13. **Gaussian distribution of mini-batch gradients under fixed parameters.** Empirical distributions of mini-batch sampling noise are shown for varying batch sizes, computed without applying updates (i.e., model parameters were held fixed throughout). This setup isolates the intrinsic gradient variability introduced solely by stochastic mini-batch sampling. The plotted distributions correspond to the gradient of a five-layer MLP trained on the MNIST dataset using vanilla stochastic gradient descent (SGD) with a learning rate of 0.01. Each distribution is fitted with a Gaussian $\mathcal{N}(0, \sigma^2)$, where σ is estimated via maximum likelihood using `scipy.stats.norm` from the SciPy library (15). As batch size increases, the variance of the mini-batch gradients systematically decreases, reflecting the averaging effect of large sample aggregates.

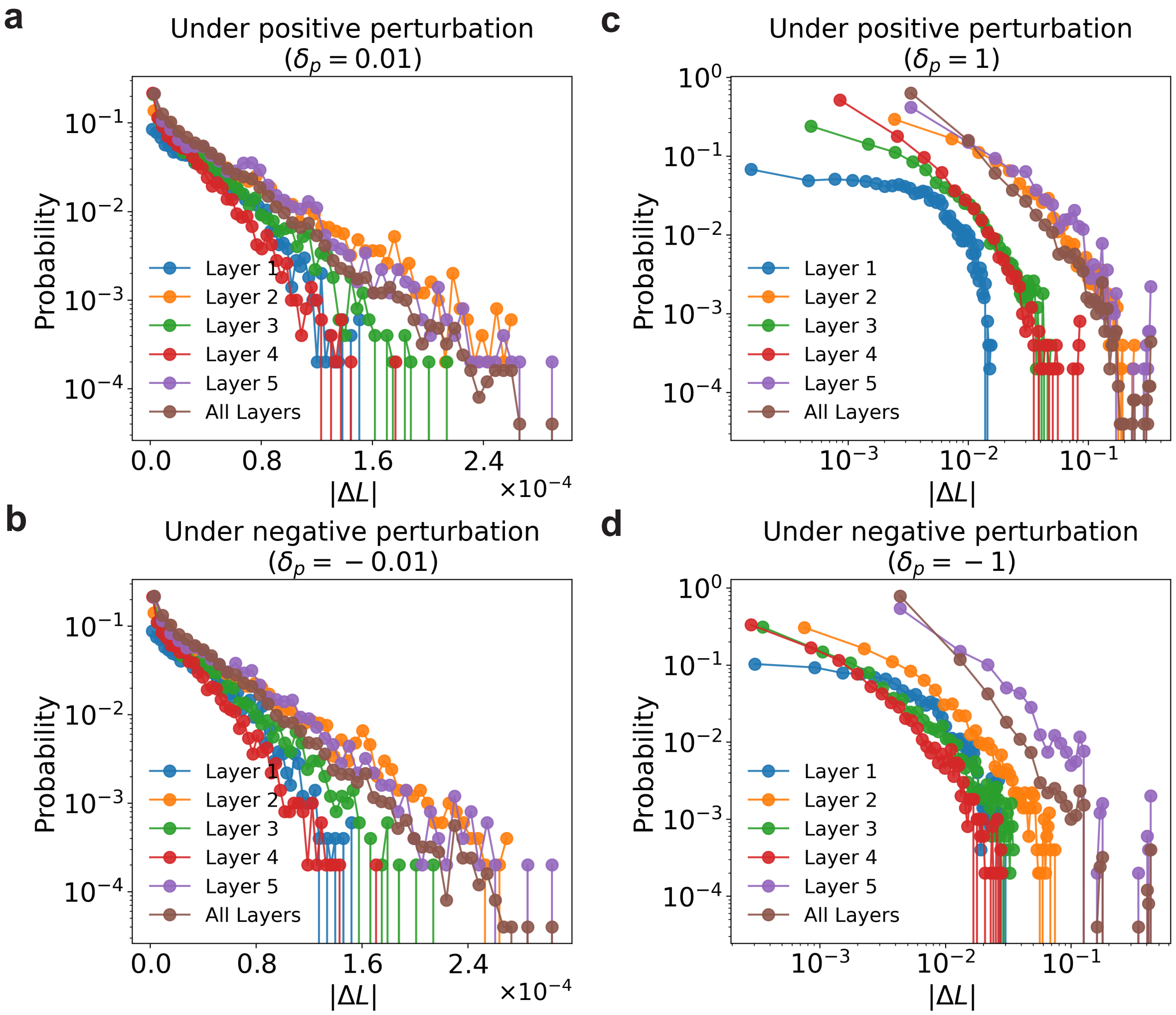

Fig. S14. **Loss sensitivity transitions from exponential to power-law across layers under increasing perturbations. a, b**, Loss change distributions $|\Delta L|$ under small perturbations ($\delta_p = \pm 0.01$) across five fully connected layers and the entire MLP model. All layers exhibit exponential-like decay, suggesting smooth local geometry within the minima basin. **c, d**, Under large perturbations ($\delta_p = \pm 1$), the distributions transition to power-law-like heavy tails, especially evident in later layers. These results highlight that the geometry of the loss landscape is scale-sensitive: it appears flat under local perturbations but exhibits power-law ruggedness at large perturbation scales.

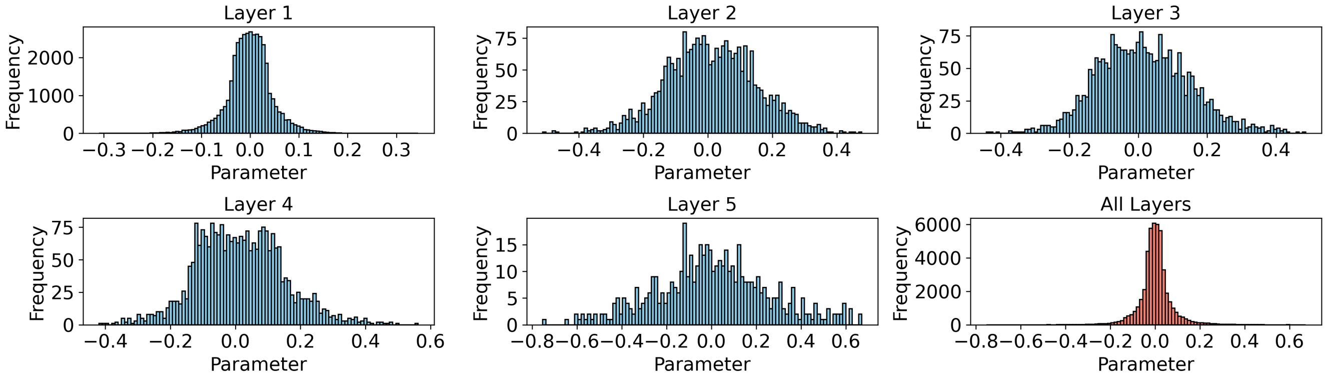

Fig. S15. **Parameter distribution across layers in a well-trained MLP.** Histogram plots show the distribution of trained parameters for each fully connected layer (1 to 5), along with the aggregated distribution for the entire network (bottom right). This empirical range provides a reference for interpreting our perturbation analysis: the small perturbation setting ($\delta_p = \pm 0.01$) probes the local ruggedness, while the large perturbation setting ($\delta_p = \pm 1$ or ± 10) represents substantially larger deviation that captures the global geometry of the loss landscape.

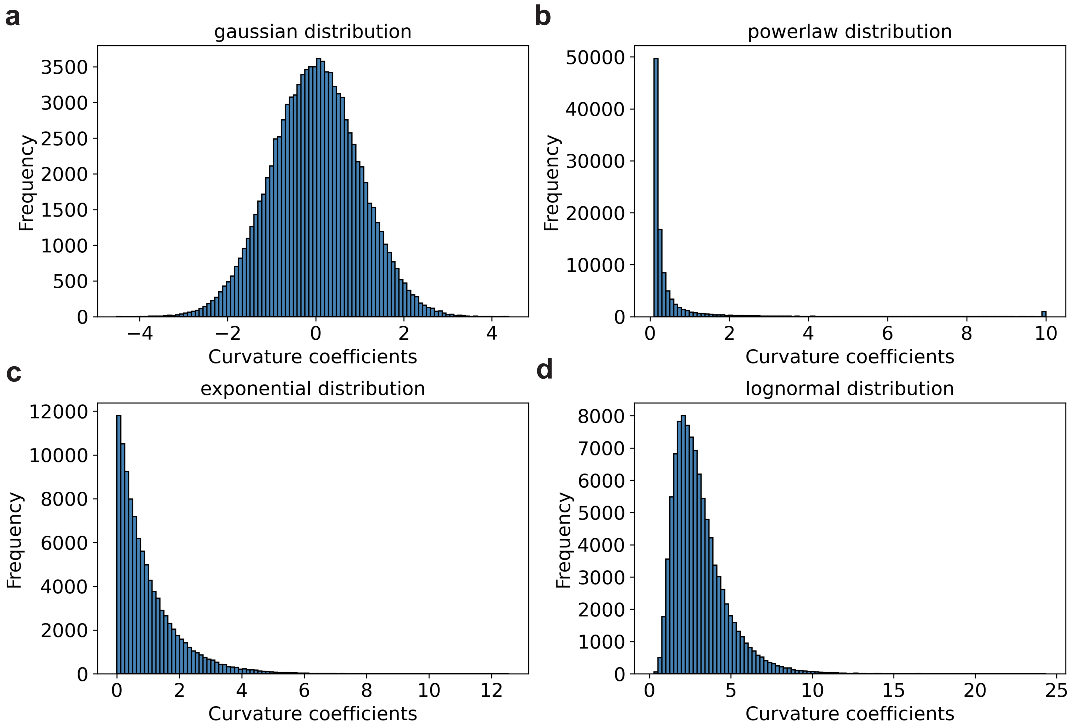

Fig. S16. **Synthetic curvature distributions for simulating loss landscape ruggedness.** Histograms of 50, 000 curvature coefficients $\{c_i\}$ used to construct synthetic high-dimensional loss landscapes with varying ruggedness. These distributions include **a**, Gaussian distribution $\mathcal{N}(0, 1)$, producing symmetric curvature centered at zero. **b**, Power-law distribution $p(c_i) \propto c_i^{-2}$, introducing heavy-tailed curvature magnitudes. **c**, Exponential distribution $p(c_i) \propto e^{-c_i}$, yielding predominantly small curvatures with rare large ones. **d**, Log-normal distribution with parameters $\mu = 1$, $\sigma = 0.5$. These predefined curvature profiles control the geometry structure of ruggedness in the synthetic loss surfaces analyzed in subsequent perturbation experiments.

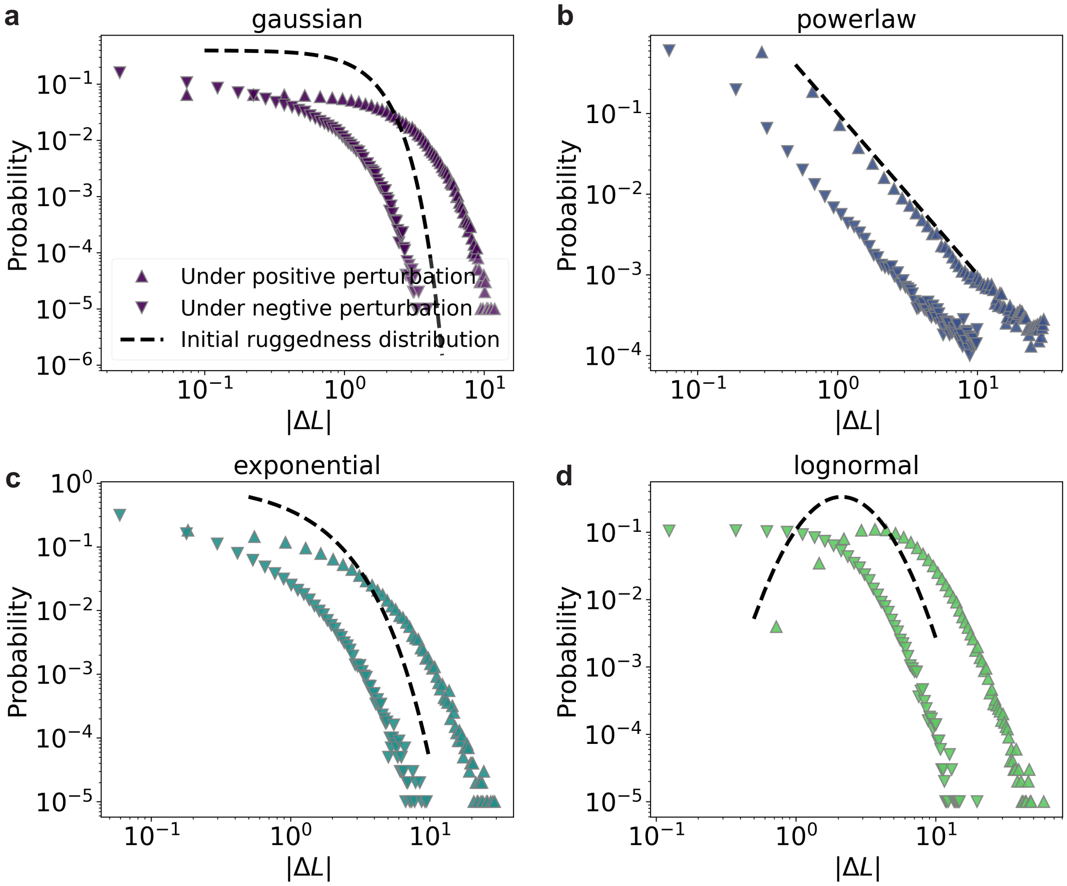

Fig. S17. **Loss perturbation response distributions under small-magnitude perturbations.** Log-scaled probability distributions of loss change magnitudes $|\Delta L|$ induced by small feature-wise perturbations $\delta_p = \pm 1$ in synthetic paraboloid landscapes with different curvature profiles. **a, d**, The curvature coefficients $\{c_i\}$ are sampled from Gaussian (a), power-law (b), exponential (c), and log-normal (d) distributions (see Fig. S16). Perturbations were applied independently along randomly chosen feature, and both positive ($\delta_p = +1$, upward triangles) and negative ($\delta_p = -1$, downward triangles) perturbations are tested. Dashed lines indicate the original statistical distribution of curvature. Loss changes under small perturbations exhibit distributions consistent with the curvature geometry, validating the effectiveness of our perturbation method in probing landscape ruggedness.

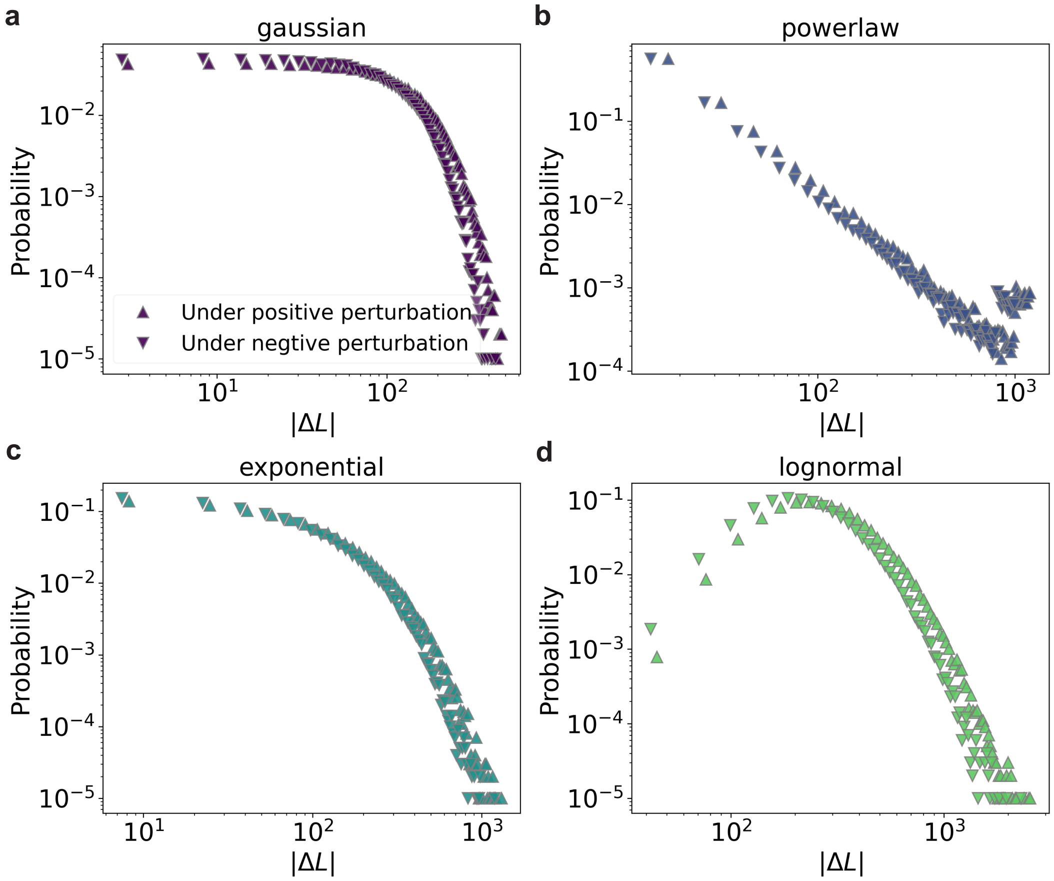

Fig. S18. **Loss perturbation response distributions under large-magnitude perturbations.** Loss change distributions $|\Delta L|$ under large feature-wise perturbations $\delta_p = \pm 10$, evaluated on the same synthetic landscapes as in Fig. S17. **a-d**, The curvature coefficients $\{c_i\}$ are drawn from Gaussian, power-law, exponential, and log-normal distributions, respectively. Compared to the small perturbation setting, the large perturbations also exhibit qualitatively similar distributional patterns, suggesting that the scale of perturbation does not fundamentally alter the underlying geometric ruggedness of the loss landscape.

References

1. Y Feng, W Zhang, Y Tu, Activity–weight duality in feed-forward neural networks reveals two co-determinants for generalization. *Nat. Mach. Intell.* **5**, 908–918 (2023).
2. H Chen, L Zheng, R Al Kontar, G Raskutti, Gaussian process parameter estimation using mini-batch stochastic gradient descent: convergence guarantees and empirical benefits. *J. Mach. Learn. Res.* **23**, 1–59 (2022).
3. A Panigrahi, R Somani, N Goyal, P Netrapalli, Non-gaussianity of stochastic gradient noise. *NeuIPS Work. on Sci. meets Eng. Deep. Learn.* (2019).
4. CM Bishop, NM Nasrabadi, *Pattern recognition and machine learning.* (Springer) Vol. 4, (2006).
5. KP Murphy, *Machine learning: a probabilistic perspective.* (MIT press), (2012).
6. DJ MacKay, *Information theory, inference and learning algorithms.* (Cambridge university press), (2003).
7. P Kadambi, KN Ramamurthy, V Berisha, Comparing fisher information regularization with distillation for dnn quantization. *NeurIPS Work. on Deep. Learn. through Inf. Geom.* (2020).
8. ET Jaynes, Information theory and statistical mechanics. *Phys. Rev.* **106**, 620 (1957).
9. T Dozat, Incorporating nesterov momentum into adam. *Int. Conf. on Learn. Represent. Work.* (2016).
10. DP Kingma, Adam: A method for stochastic optimization. *Int. Conf. on Learn. Represent.* (2014).
11. R Elshamy, O Abu-Elnasr, M Elhoseny, S Elmougy, Improving the efficiency of rmsprop optimizer by utilizing nestrove in deep learning. *Sci. Reports* **13**, 8814 (2023).
12. J Duchi, E Hazan, Y Singer, Adaptive subgradient methods for online learning and stochastic optimization. *J. Mach. Learn. Res.* **12** (2011).
13. X Glorot, Y Bengio, Understanding the difficulty of training deep feedforward neural networks. (JMLR Workshop and Conference Proceedings), pp. 249–256 (2010).
14. K He, X Zhang, S Ren, J Sun, Delving deep into rectifiers: Surpassing human-level performance on imagenet classification. *Proc. IEEE Int. Conf. on Comput. Vis.* pp. 1026–1034 (2015).
15. P Virtanen, , et al., SciPy 1.0: Fundamental Algorithms for Scientific Computing in Python. *Nat. Methods* **17**, 261–272 (2020).
